# Tin-graphene tubes as anodes for lithium-ion batteries with high volumetric and gravimetric energy densities

**DOI:** 10.1038/s41467-020-14859-z

**Published:** 2020-03-13

**Authors:** Runwei Mo, Xinyi Tan, Fan Li, Ran Tao, Jinhui Xu, Dejia Kong, Zhiyong Wang, Bin Xu, Xiang Wang, Chongmin Wang, Jinlai Li, Yiting Peng, Yunfeng Lu

**Affiliations:** 1 Chemical and Biomolecular Engineering, University of California, Los Angeles, CA 90095 USA; 2 State Key Laboratory of Supramolecular Structure and Materials, Jilin University, 130012 Changchun, China; 3 Environmental Molecular Sciences Laboratory, Pacific Northwest National Laboratory, Richland, WA 99352 USA; 4 ENN Group, Langfang, 065001 Hebei, China; 5 Shanghai Key Laboratory of Materials Protection and Advanced Materials in Electric Power, Shanghai University of Electric Power, 200090 Shanghai, China

**Keywords:** Batteries, Batteries, Batteries

## Abstract

Limited by the size of microelectronics, as well as the space of electrical vehicles, there are tremendous demands for lithium-ion batteries with high volumetric energy densities. Current lithium-ion batteries, however, adopt graphite-based anodes with low tap density and gravimetric capacity, resulting in poor volumetric performance metric. Here, by encapsulating nanoparticles of metallic tin in mechanically robust graphene tubes, we show tin anodes with high volumetric and gravimetric capacities, high rate performance, and long cycling life. Pairing with a commercial cathode material LiNi_0.6_Mn_0.2_Co_0.2_O_2_, full cells exhibit a gravimetric and volumetric energy density of 590 W h Kg^−1^ and 1,252 W h L^−1^, respectively, the latter of which doubles that of the cell based on graphite anodes. This work provides an effective route towards lithium-ion batteries with high energy density for a broad range of applications.

## Abstract (web summary)

Here the authors report a tin anode design by encapsulating tin nanoparticles in graphene tubes. The design exhibits high capacity, good rate performance and cycling stability. Pairing with NMC, the full cell delivers a volumetric energy density twice as high as that for the commercial cell.

## Introduction

There are increasing demands for lithium-ion batteries (LIBs) with high gravimetric energy; meanwhile, due to the limited space that are available to accommodate the batteries in microelectronics and electric vehicles, developing LIBs with high volumetric energy density is also emerging as a particularly important theme^1–5^. The current LIBs generally adapt graphite as the anode material and lithium-nickel-manganese-cobalt oxides (NMC) as the cathode materials. Graphite has a theoretical gravimetric capacity of 372 mA h g^**−**1^ (based un-lithiated graphite), crystal density of 2.266 g cm^**−**3^, and volumetric capacity of 841 mA h cm^**−**3^ (based on un-lithiated graphite)^6^ or 719 mA h cm^**−**3^ (based on full-lithiation graphite)^7^. Whereas the commercially used graphite generally has a low tap density (e.g., 1.1 g cm^**−**3^), which occupies a significant portion of a battery volume^8^. For example, when paired with commercial cathode materials (e.g., NMC111, NMC523, NMC622, and NCM811), the graphite coatings could account for 55–60% of the volume of a cell, including the anode and cathode coatings, current collector, and separator^9–11^. Exploring novel anode materials that can reduce the volume of the anode coatings occupied, in this context, is of paramount importance towards LIBs with significantly improved volumetric energy density.

Among the vast library of anode materials, metals and metal oxides generally exhibit significantly higher volumetric capacities than the carbonaceous materials owning to their high gravimetric capacity and tap density^12–15^. For example, tin (Sn) has a theoretical volumetric capacity of 7316 mA h cm^**−**3^ (based on un-lithiated Sn)^16–18^ or 2111 mA h cm^**−**3^ (based on full-lithiation Sn)^7^, respectively, which are significantly higher than those of graphite (841 mA h cm^**−**3^ and 719 mA h cm^**−**3^, respectively)^7,16–18^. In addition, Sn is a low-cost material with a low working potential (<0.5 V versus Li/Li^+^), making it a highly promising anode candidate with both high volumetric and gravimetric energy density. In fact, Sn was commercially used as the anodes for Nexelion made by Sony^19,20^. Nonetheless, Sn exhibits large-volume change during the lithiation and delithiation, which disrupts the electrode structure and electronic conductive networks and results in poor cycling life^19,20^. To address these issues, various Sn, Sn alloys^21–25^, and Sn-based composites^26–35^ with designed structures (e.g., nanowires, nanosheets, and porous structures)^36–40^ and compositions have been explored, whereas making Sn anodes with high energy density and long cycling life remains challenging.

**Fig. 1:**
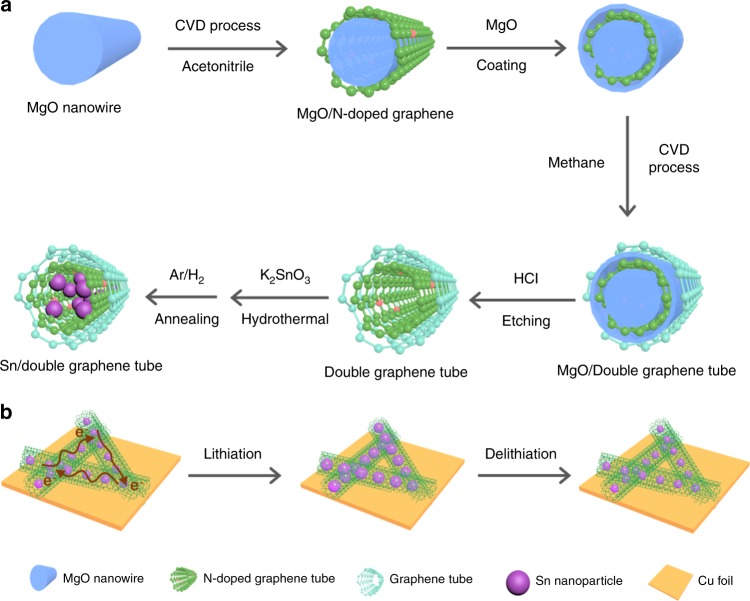
A schematic illustrating the synthesis of Sn/DGT for high-performance anodes. **a** A schematic illustration of the synthesis of Sn/DGT composite. **b** A schematic illustration of the lithiation process of a Sn/DGT electrode, during which Sn nanoparticles are retained within the graphene tubes despite their large-volume change, preserving the electron-conduction networks and integrity of the electrode.

We show a design of high-performance Sn anodes, which were made by confining Sn nanoparticles within the frameworks of graphene tubes. As illustrated in the Fig. 1a, using magnesium oxide (MgO) as the template and catalyst, we first grew nitrogen-doped graphene around MgO nanowires by chemical vapor deposition (CVD) using acetonitrile as the precursor. As-formed graphene-coated nanowires were then coated with a thin layer of MgO, on which graphene was grown using methane as the precursor (Supplementary Fig. 1). Removal of the templates leads to the formation of double-graphene-tubes (DGT), which consists of an inner hydrophilic graphene tube (nitrogen-doped) and an outer hydrophobic graphene tube (un-doped). Dispersing the DGT in a K_2_SnO_3_ solution allows the aqueous precursor to infiltrate into the hydrophilic tubes, as well as growth of SnO_2_ nanoparticles within the inner tubes in a subsequent hydrothermal reaction. Finally, reducing the SnO_2_ nanoparticles leads to the formation of Sn nanoparticles encapsulated within the double-graphene-tubes, denoted as Sn/DGT.

As depictured in the Fig. 1b, by confining the Sn nanoparticles within the electronically conductive and mechanically robust DGT, the structural integrity of the electrodes, as well as the electron-conduction networks among the nanoparticles, could be well preserved, despite their large-volume change during the lithiation and delithiation. In addition, the small size of the Sn nanoparticles shortens their ion-diffusion paths and alleviates the mechanical stress during the alloying/dealloying process. Meanwhile, the tubular structure allows a high loading of Sn nanoparticles, which is critical for high energy density. Note that Sn-carbon composites were also synthesized by infiltrating Sn precursors to carbon scaffolds (e.g., porous carbon particles and carbon fibers) followed by a reduction process^26–35^. In these approaches, it is difficult to assure as-formed Sn particles are confined within the scaffolds, whereas detachment of the Sn particles from the carbon scaffolds could unavoidably result in decay of the capacity.

Here in this work, by confining a high loading of Sn nanoparticles within highly conductive DGT, our strategy avoids the detachment of the Sn nanoparticles from the conductive networks, affording the anodes with high volumetric capacity, outstanding power performance and long cycling life. As-made Sn anodes can provide high capability (918 mA h g^**−**1^ or 2532 mA h cm^**−**3^), long cycling stability (>95% reversible capacity retention after 500 cycles), and excellent rate performance (402 mA h g^**−**1^ at current density 20 A g^**−**1^). When paired with NCM622, full cells with high energy density (598 W h Kg^**−**1^ or 1252 W h L^**−**1^) are demonstrated, which are 1.4 and 1.9 times higher than those made with commercial graphite anodes, respectively.

## Results

### Materials synthesis and characterization

**Fig. 2:**
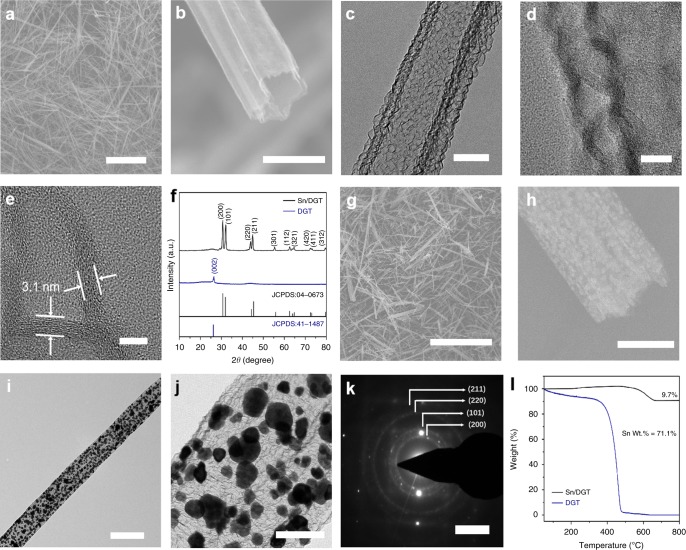
Morphology and structure of DGT and Sn/DGT. **a**, **b** SEM images of DGT. Scale bars: **a** 10 μm; **b** 500 nm, **c**–**e** TEM images of DGT. Scale bars: **c** 200 nm; **d** 10 nm; **e** 5 nm, **f** XRD patterns of DGT and Sn/DGT, **g**, **h** SEM images of Sn/DGT. Scale bars: **g** 10 μm; **h** 200 nm, **i**, **j** TEM images of Sn/DGT. Scale bars: **i** 500 nm; **j** 100 nm, **k** selective area electronic diffraction of Sn/DGT. Scale bars: **k** 5 1/nm, and **l** TGA of DGT and Sn/DGT.

Figure 2a–d shows the scanning electron microscopic (SEM) and transmission electron microscopic (TEM) images of DGT, which show an average diameter of 350 nm and a length between 10 μm and 20 μm (Fig. 2a). The double-tube structure can be clearly visualized from the SEM image shown in Fig. 2b, which is further confirmed by the TEM image in Fig. 2c. The distance between the inner and outer tube is ~10 nm; meanwhile, porous structure in the tube wall can be clearly observed, providing pathways for the transport of electrolytes (Fig. 2d). Figure 2e shows a higher-magnification TEM image of DGT, indicating the graphene structure has a tube-wall thickness of 3.1 nm (approximately nine layers of graphene). In addition, chemical mapping of the graphene tubes confirms the presence of nitrogen, indicating successful nitrogen doping of the inner graphene tubes (Supplementary Fig. 2).

Figure 2f shows the X-ray diffraction (XRD) of DGT and Sn/DGT. DGT exhibits the (002) peak at ~26°, Sn/DGT exhibits intense diffractions of tetragonal Sn (JCPDS No. 04-0673)^39,40^, and no diffraction peaks from the graphene tubes can be observed due to its low content. Raman spectra of Sn/DGT show two peaks centered at 1331 cm^–1^ and 1586 cm^–1^, attributed from the DGT moiety (Supplementary Fig. 3)^41,42^. Figure 2g, h shows the SEM images of Sn/DGT, where Sn nanoparticles are encapsulated within the graphene tubes. TEM image suggests that Sn nanoparticles are uniformly distributed within the DGT (Fig. 2i) with an average diameter of 40 nm (Fig. 2j). No Sn nanoparticles outside the DGT could be observed. As demonstrated in Supplementary Fig. 4, the Sn nanoparticles have an interplanar distance of 0.29 nm, attributed to the (200) facet of tetragonal Sn^39,40^. Figure 2k exhibits the selected-area electron diffraction (SAED) of Sn/DGT confirming the formation of tetragonal-phase Sn. Figure 2l shows the thermal gravimetric analysis (TGA) of DGT and Sn/DGT measured using an air atmosphere. DGT experiences a major weight loss from 400 to 500 °C due to its oxidation reaction, while the weight of Sn/DGT increases before 500 °C attributed to the oxidation of the Sn nanoparticles. Further increasing the temperature causes oxidation of the DGT moiety, accompanied by a weight loss. The final weight loss of Sn/DGT is around 9.7%, corresponding to a Sn content of ~71.1 wt%.

**Fig. 3:**
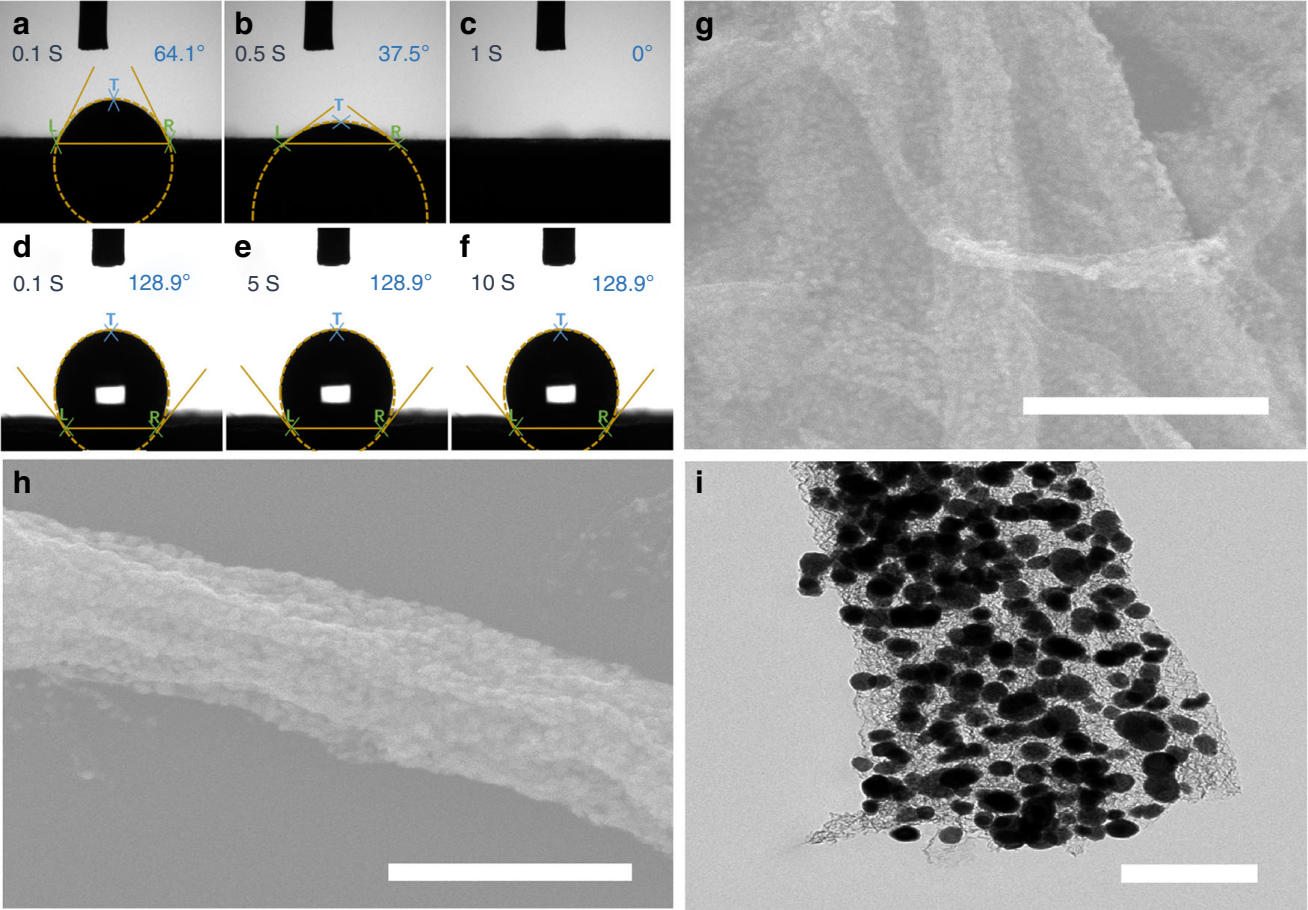
Wetting process of water on nitrogen-doped and un-doped graphene tubes and the role of biphilicity on the selective growth of SnO_2_. Wetting process of water on nitrogen-doped graphene tubes (a–c) and un-doped graphene tubes (**d**–**f**). **g**, **h** SEM and i TEM images of Sn/DGT*. Scale bars: **g** 2 μm; **h** 500 nm; **i** 200 nm.

Figure 3a–f shows a wetting process of water on nitrogen-doped (A-C) and un-doped (D-F) graphene tubes. The nitrogen-doped graphene tubes (the inner tubes of DGT) exhibit an initial contact angle of 64.1°, which is rapidly decreased to 0° after 1 s, indicating a hydrophilic surface. In contrast, the un-doped graphene tubes (the outer tubes of DGT) show a stable contact angle of ~128.9°, indicating a hydrophobic surface. Such biphilicity (the co-exitance of hydrophilic and hydrophobic tubes) is critical to ensure the encapsulation of Sn nanoparticles within the DGT and avoid their attachment outside the DGT.

Firstly, the biphilicity of DGT allows the aqueous precursor solution to infiltrate to the inner hydrophilic tubes, where tin oxide (SnO_2_) nanoparticles with a tetragonal rutile SnO_2_ phase (JCPDS No. 41–1445) were formed (Supplementary Fig. 5). Supplementary Fig. 6 shows the SEM and TEM images of a SnO_2_/DGT composite. The graphene tubes maintain their tubular structure after the growth of SnO_2_ nanoparticles (Supplementary Fig. 6a); chemical mapping indicates a uniform distribution of the nanoparticles within the DGT (Supplementary Fig. 6b). Secondly, it appears that Sn nanoparticles prefer to grow on nitrogen-doped graphene surface than the un-doped graphene surface. To confirm, we prepared double-graphene-tubes that consist an outer hydrophilic graphene tube and an inner hydrophobic graphene tube. Sn nanoparticles were also grown on these tubes using a similar process, resulting in a composite denoted as Sn/DGT* (Supplementary Figs. 7, 8). As expected, an appreciable amount of Sn nanoparticles was grown outside the double-graphene-tubes (Fig. 3g–i), confirming the roles of biphilicity on selective growth of SnO_2_ nanoparticles within the DGT. Thirdly, although Sn nanoparticles may also be formed in the reaction solution; such nanoparticles could be readily removed during the filtration process, a step used to separate Sn/DGT from the reaction mixture. The ability to confine the growth of Sn nanoparticles within DGT, as well as the ability to remove free Sn nanoparticles from the Sn/DGT, is essential to ensure the high performance of such Sn anodes. For further confirmation, hydrophobic graphene tubes were also synthesized, and the growth of Sn nanoparticles on the hydrophobic graphene tubes could not be observed (Supplementary Fig. 9).

### Electrochemical performance

**Fig. 4:**
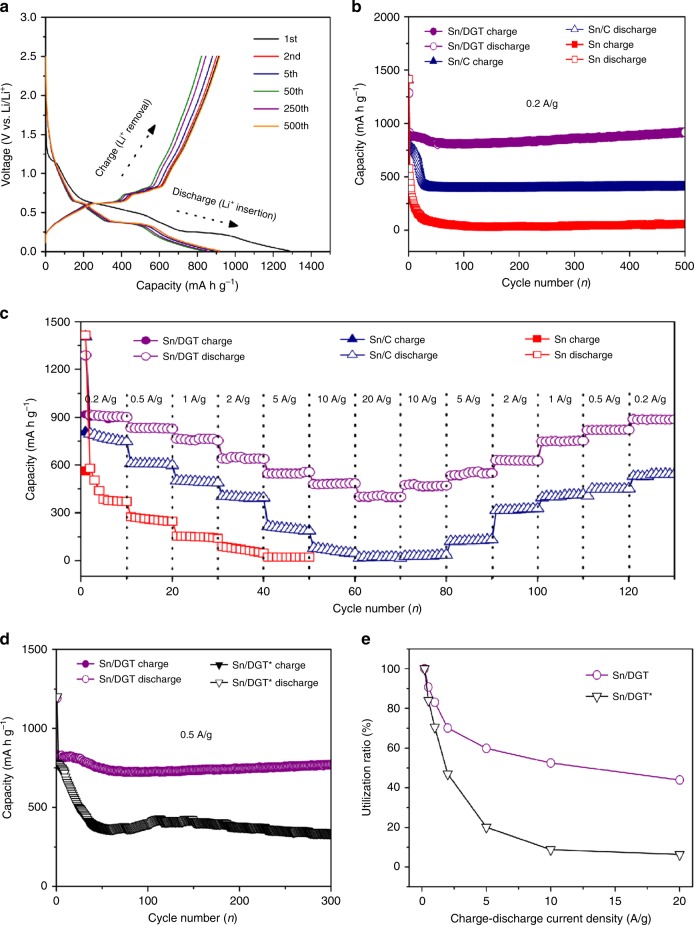
Electrochemical performance of the Sn/DGT. **a** Galvanostatic charge-discharge profiles of Sn/DGT from 0.01 to 2.5 V (versus Li/Li^+^) for the 1st, 2nd, 10th, 100th, and 500th cycles at a current density of 0.2 A g^**−**1^. **b** Cycling performance (charge/discharge) of the Sn/DGT electrode, Sn/C and Sn electrodes with a mass loading of 2 mg cm^**−**2^ at a current density of 0.2 A g^**−**1^ for 500 cycles, respectively. **c** The capacity of the Sn/DGT electrode, Sn/C and Sn electrodes at different current densities. **d** Cycling performance (charge/discharge) of the Sn/DGT electrode and Sn/DGT* electrode with a mass loading of 2 mg cm^**−**2^ at a current density of 0.5 A g^**−**1^ for 300 cycles. **e** Utilization of the active material of the Sn/DGT electrode and Sn/DGT* electrode at different charge-discharge current densities.

Figure 4a shows the representative charge/discharge voltage profiles of a Sn/DGT electrode from 0.01 to 2.5 V (vs. lithium metal) at 0.2 A g^**−**1^. The electrode shows an initial discharge and charge capacity of 1285 mA h g^**−**1^ and 913 mA h g^**−**1^, corresponding to an initial Coulombic efficiency of 71.1%. The excess discharge capacity could be attributed to the decomposition of the electrolyte, the formation of solid electrolyte interphase (SEI), and to irreversible insertion of Li ions into the Sn^34–37^. The capacity of the Sn/DGT electrode is mainly contributed by Li insertion at voltage below 0.5 V (vs. Li^+^/Li), which ensures a high full-cell voltage and high energy density^43^.

As shown in Fig. 4a, the electrode retains a capacity of 918 mA h g^**−**1^ after 500 cycle, indicating an excellent cycling stability. To further assess the cycling stability of Sn/DGT, electrodes made from commercial Sn particles, as well as a Sn-carbon composite (denoted as Sn/C) was also examined. The Sn/C was synthesized by polymerizing dopamine-hydrochloride on commercial Sn particles followed by carbonization (Supplementary Figs. 10–12). Figure 4b compares the capacity of the electrodes made from Sn/DGT, Sn/C, and Sn particles, respectively. After cycling for 500 cycles at a current density of 0.2 A g^**−**1^, the Sn/DGT electrode still retains a high Coulombic efficiency (>99%) and a high capacity of 916 mA h g^**−**1^, which is 4-folds and 16-folds higher than that of the Sn/C (201 mA h g^**−**1^) and Sn (58 mA h g^**−**1^) electrode, respectively. Meanwhile, the capacity of the Sn/DGT electrode increases with time possibly due to a continuous activation process^36–38^.

The rate capability of the Sn/DGT electrodes were evaluated at the current densities of 0.2, 0.5, 1, 2, 5, and 10 A g^**−**1^, which provides a capacity of 916, 831, 761, 642, 548, and 481 mA h g^**−**1^, respectively (Fig. 4c). Even with a higher current density of 20 A g^**−**1^ (~20 C), the electrode can still provide a remarkably high capacity of 402 mA g h^**−**1^. Returning the cycling current density to 0.2 A g^**−**1^, the capacity is recovered to 913 mA g h^**−**1^, indicating an outstanding rate capability^44^. The capacity of the composite is contributed by the Sn and DGT moieties. At a voltage window of 0.01–2.5 V, a DGT electrode exhibits a capacity of 712, 665, 592, 551, 474, 371, and 252 mA h g^**−**1^ at the current density of 0.2, 0.5, 1, 2, 5, 10, and 20 A g^**−**1^, respectively (Supplementary Fig. 16). Considering the Sn/DGT composite contains 29 wt% graphene, the capacity contributed by DGT can be estimated as 206, 192, 172, 160, 137, 108, and 73 mA h g^**−**1^, respectively. Such SEI layer could be partly decomposed during the charge process, contributing to the charge capacity^45^. And the other is the improved lithium storage capacity by the synergetic effect between conducting DGT and Sn NPs, which is responsible for the excellent electrochemical performance of the overall electrode via the maximum utilization of electrochemically active DGT and nanosized Sn^31,46^.

The outstanding rate capability can be attributed to the excellent electrical conductivity and fast diffusion of lithium ions^47^. For further confirmation, Supplementary Fig. 13 compares the Nyquist plots of the Sn/DGT, Sn/C, and Sn electrodes, where the Sn/DGT electrode exhibits a much smaller resistance than the others. Supplementary Fig. 14 provides an equivalent circuit, which consists of a solution resistance (R_s_), a charge-transfer resistance (R_ct_), a contact resistance (R_f_), and a Warburg impedance (Z_w_). As depicted in Supplementary Table 1, the calculated R_ct_ and R_f_ values of the Sn/DGT electrode are significantly lower than those of the Sn/C and Sn electrodes. It is interesting to compare the performance of Sn/DGT electrodes with and without carbon black additive. It was found that the electrode without carbon black shows a capacity of 907 mA h g^**−**1^ at 0.2 A g^**−**1^, whereas increasing the current density deteriorates the rate performance (Supplementary Fig. 15). This result suggests that the use of nanosized carbon black is still necessary in order to construct highly effective conductive networks^40^, despite the excellent electrical conductivity of the DGT.

The outstanding cycling stability and rate performance are contributed from the ability to encapsulate the Sn nanoparticles within the highly conductive and robust graphene tubes. Figure 4d compares the cycling stability of Sn/DGT and Sn/DGT* (mass loading of 2 mg cm^**−**2^) at current density of 0.5 A g^**−**1^, which show a similar initial discharge and charge capacity of 1200 mA h g^**−**1^ and 810 mA h g^**−**1^. However, Sn/DGT* contains a significant amount of Sn nanoparticles outside the graphene tubes (see Fig. 3), which can be easily detached from the graphene tubes during the cycling, resulting in a capacity decay to 337 mA h g^**−**1^ after 300 cycles. Whereas Sn/DGT still retains a high capacity of 769 mA h g^**−**1^, confirming the encapsulation of the Sn nanoparticles do critically contribute to the cycling stability. Supplementary Fig. 17 shows TEM images of the Sn/DGT anode after cycling at 5 A g^**−**1^ for 500 cycles, confirming that the Sn nanoparticles were still confined within the graphene tubes. The electrode exhibits the first-cycle discharge and charge capacities of 536 and 722 mA h g^**−**1^ at the current density of 5 A g^**−**1^, respectively, giving a first-cycle Coulombic efficiency of 74.1% (Supplementary Fig. 17a), which increases to greater than 98% in the second cycle and to around 99.9% after 500 cycles (the high first-cycle capacity is probably due to SEI formation). Note that the accurate measurement of the Coulombic efficiency of half cells is a critical factor to predict the cycle life of full cells. Figure 4e further plots the utilization of the active material versus the charge-discharge current density of the Sn/DGT and Sn/DGT* electrodes. The utilizations are estimated by normalizing the specific capacity of the electrodes at different charge-discharge current densities (the slopes of the lines) vs. the specific capacity at 0.2 A g^**−**1^. As shown, the active-material utilization decreases with increasing charge-discharge current density, which is 60%, 52%, and 43% for the Sn/DGT electrode and 20%, 9%, and 6 % for the Sn/DGT* electrode at 5, 10, and 20 A g^**−**1^, respectively. To further assess the performance of the Sn/DGT electrodes, a comparison between the Sn/DGT electrode and other Sn electrodes reported is also provided (Supplementary Table 2).

**Fig. 5:**
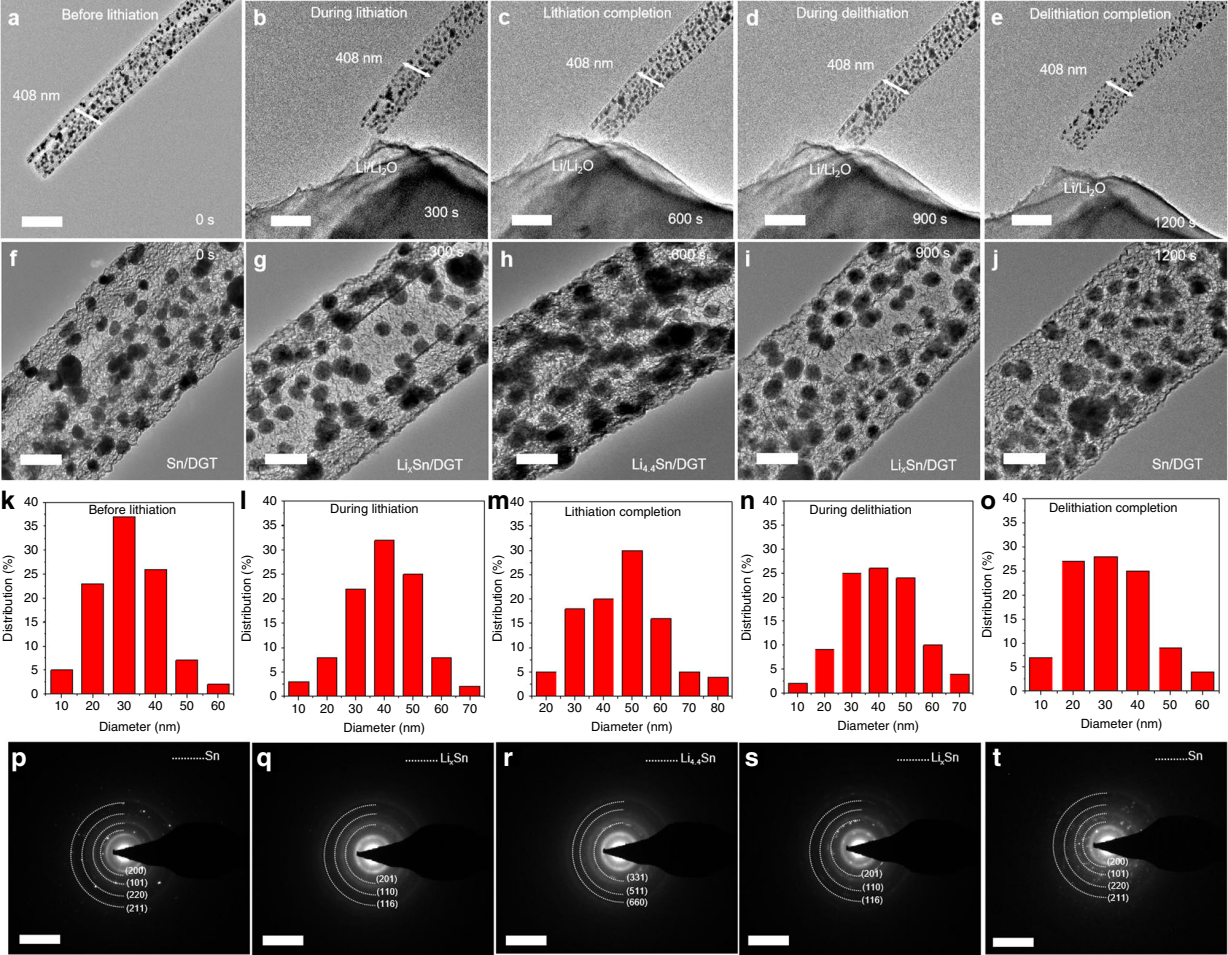
In situ TEM observation of Sn/DGT during a lithiation-delithiation cycle. **a**–**e** Time-lapse low magnification and **f**–**j** high-magnification TEM images showing the lithiation and delithiation of a Sn/DGT electrode (Supplementary Movies 1, 2). During this process, the Sn nanoparticles experienced volume expanding and shrinking, yet remained within the graphene tube, of which the structure and dimension remains unchanged. Scale bars: **a** 500 nm; **b** 500 nm; **c** 500 nm; **d** 500 nm; **e** 500 nm; **f** 100 nm; **g** 100 nm; **h** 100 nm; **i** 100 nm; **j** 100 nm. **k**–**o** Size analysis of the Sn nanoparticles during the lithiation-delithiation cycle obtained using a semi-automated sizing approach. **p**–**t** The selective area electron diffraction patterns of the Sn nanoparticles during the lithiation-delithiation cycle. Scale bars: **p** 5 1/ nm; **q** 5 1/nm; **r** 5 1/nm; **s** 5 1/nm; **t** 5 1/nm.

To further probe the outstanding electrode performance, the lithiation and delithiation process of Sn/DGT were probed by in situ TEM (Supplementary Fig. 18 and Supplementary Movies 1, 2). Figure 5 shows the low-magnification (a–e) and high-magnification (f–j) TEM images of a Sn/DGT tube during the lithiation and delithiation. The diameter of the graphene tube remains unchanged (480 nm), whereas the Sn nanoparticles reversibly expand and shrink during the lithiation and delithiation, of which the change of the particle size are plotted in Fig. 5k–o, respectively. As shown, despite the volume change, these particles remain confined within the graphene tubes. Figure 5p–t shows the selective area electronic diffraction of Sn/DGT during this process. The Sn nanoparticles exhibit a tetragonal crystal structure (*a* = 5.8316 Å, *c* = 3.1813 Å, space group 141) prior to the lithiation (Fig. 5p). After lithiation for 300 s, the diffraction spots are changed to diffraction rings, consistent with a polycrystalline Li_x_Sn phase. The rings corresponding to a *d*-spacing of 0.192 nm and 0.237 nm are likely attributable to Li_5_Sn_2_, while the ring corresponding to a *d*-spacing of 0.327 nm is likely attributable to Li_7_Sn_3_ phase (Fig. 5q). After lithiation for 600 s, the Li_x_Sn phase was converted to the Li_4.4_Sn phase, corresponding to the diffraction rings with the *d*-spacings of 0.232, 0.379, and 0.452 nm (Fig. 5r). The subsequent delithiation process reverses the Li_4.4_Sn phase sequentially to Li_x_Sn (Fig. 5s) and to tetragonal Sn (Fig. 5t). This observation confirms a reversible phase transformation of the Sn nanoparticles during the lithiation and delithiation, which are confined with the highly conductive and robust graphene tubes and leads to the outstanding cycling stability and rate performance observed.

**Fig. 6:**
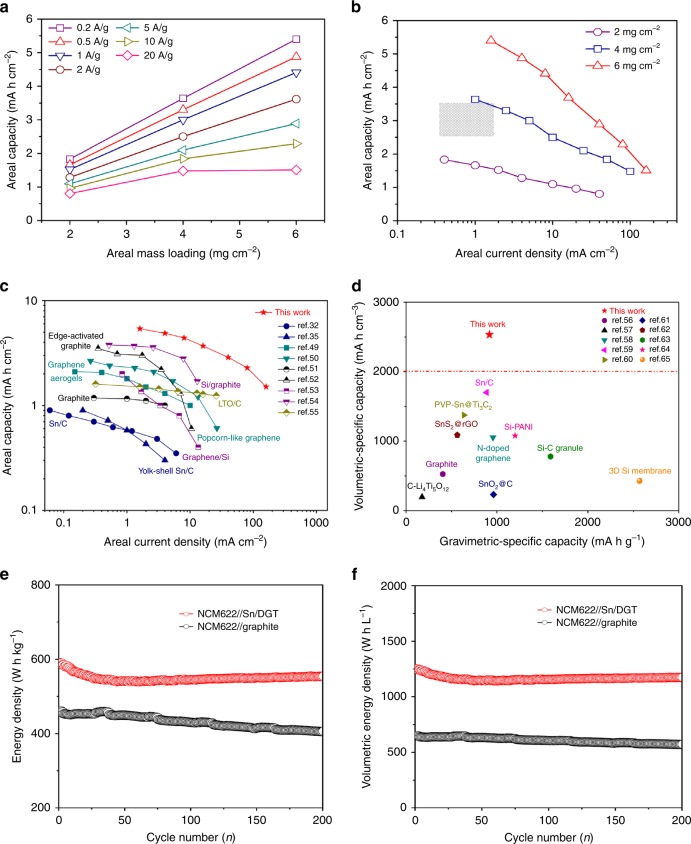
Feasibility of adapting Sn/DGT as high-performance anodes for commercial batteries. **a** The areal capacity of Sn/DGT electrodes with mass loading of 2, 4, and 6 mg cm^**−**2^ at different charge-discharge current densities. **b** The areal capacity of Sn/DGT electrodes vs. the areal current density of the Sn/DGT anodes with a mass loading of 2, 4, and 6 mg cm^**−**2^, respectively. The gray area marked represents the range of areal capacity of commercial graphite anodes. **c** A comparison of the areal performance metrics of Sn/DGT electrode (mass loadings of 6 mg cm^**−**2^) with representative anodes reported, including the anodes from graphite, graphene, Li_4_Ti_5_O_12_ (LTO), Sn/C and Si/C. **d** A comparison of the specific volumetric capacity of Sn/DGT electrode (active materials only) with reported anode materials, including the anodes of graphite, LTO, Sn/C, and Si/C. **e** The gravimetric and **f** volumetric energy density of a cell consisting with a NCM622 cathode and a Sn/DGT anode, as well as a cell consisting with a NCM622 cathode and graphite anode.

To evaluate that feasibility of adapting Sn/DGT for commercial use, electrodes with different areal mass loadings were fabricated by changing the thickness of the electrode. Figure 6a shows the areal capacity *vs*. the areal mass loading of the electrodes at different current densities. The areal capacity increases linearly with the areal mass loading at relatively low current densities (e.g., <5 A g^**−**1^); whereas further increasing the current density deviates the linear relation. Figure 6b plots the areal capacity vs. the areal current density, where the electrode with a mass loading of 6 mg cm^**−**2^ exhibits an areal capacity of 5.4, 4.9, 4.4, and 3.7 mA h cm^**−**2^ at the areal current density of 1.6, 4, 8, and 16 mA cm^**−**2^, respectively. For comparison, commercial graphite anodes generally provide areal capacities in the range of 2.5–3.5 mA h cm^**−**2^ at a current-density range of 0.3–1.86 mA cm^**−**2^ (see the marked area marked in Fig. 6b)^48^. Clearly, Sn/DGT electrodes well outperform the commercial graphite anodes. Furthermore, these thick Sn/DGT electrodes also exhibit outstanding cycling stability. For example, after cycling a current density of 2 A g^**−**1^ for 200 cycles, these electrodes retain over 90% of their initial capacity (Supplementary Fig. 19a) with low charge-transfer resistance indicated by their electrochemical impendence spectra (Supplementary Fig. 19b).

Figure 6c plots the areal capacity vs. the areal current density of representative anode materials, including commercial graphite, graphene, Li_4_Ti_5_O_12_, Sn/C, and Si/C-based composites. As shown, graphene aerogel, popcorn-like graphene^49–51^, and edge-activated graphite exhibit higher areal capacities and improved rate performance that graphite. Incorporating high-capacity Si to graphene and graphite further increases their areal capacity at low current density. However, inherently limited by the slow reaction between silicon and lithium, the capacity is decreased rapidly with increasing the current density. The Sn/DGT electrodes well outperformances the reported electrodes in both areal capacity and rate performance. For example, the Sn/DGT anode can supply a high areal capacity over 4 mA h cm^**−**2^ at the areal current density of 10 mA cm^**−**2^, which is the best-known values for the anodes reported^32,35,41,49–54^.

Figure 6d compares the volumetric capacities of representative anode materials vs. their gravimetric capacity, including commercial graphite, Li_4_Ti_5_O_12_, Sn-based, and Si-based anodes reported^12,55–63^. The volumetric capacities of the anode materials (active material only) were estimated based on the tap density and gravimetric specific capacity in un-lithiated state. Sn/DGT shows a tap density around 2.76 g cm^**−**3^, corresponding a volumetric capacity of 2532 mA h cm^**−**3^ and 1106 mA h cm^**−**3^ for the Sn/DGT composite at a current density of 0.2 A g^**−**1^ and 20 A g^**−**1^, respectively (based on the tap density) (Supplementary Fig. 20). The electrode retains a high volumetric capacity of 2528 mA h cm^**−**3^ after 500 cycles under a current density of 0.2 A g^**−**1^ (Supplementary Fig. 21). The volumetric capacity of the Sn/DGT electrodes well outperformances the reported anodes, despite their gravimetric capacity is less than the silicon granule^12^ and 3D silicon membrane^63^.

Note that it is more accurate and meaningful to present the volumetric capacity of anode materials or electrodes in full-lithiation state^7^. However, due to the lack of data in specific density of the anode materials that are in full-lithiation state, it is difficult to compare the volumetric capacity of the anode materials in full-lithiation state. Meanwhile, most literatures did not provide the thickness of the electrodes (un-lithiated or lithiated state), making it difficult to compare the volumetric capacity of the whole electrodes. To be more accurate, the thickness of the Sn/DGT electrodes (mass loading of 2, 4, and 6 mg cm^**−**2^) was measured before and after lithiation using SEM (Supplementary Fig. 22). The electrodes experience a thickness change of ~18% upon lithiation and well retain their structure integrity. Under a current density of 0.2 A g^**−**1^, these electrodes before and after lithiation exhibit a volumetric capacity of 1146 mA h cm^**−**3^ and 963 mA h cm^**−**3^ with a mass loading of 2 mg cm^**−**2^, 1210 mA h cm^**−**3^, and 1037 mA h cm^**−**3^ with a mass loading of 4 mg cm^**−**2^, and 1197 mA h cm^**−**3^, and 1017 mA h cm^**−**3^ with a mass loading of 6 mg cm^**−**2^, respectively. Such volumetric capacities are comparable to the record high volumetric value of Sn-based electrodes (Supplementary Table 3).

Finally, to demonstrate the viability of using Sn/DGT as the high-performance anodes, full cells were assembled using a commercial cathode material, lithium-nickel cobalt manganese oxide (NCM622) with a bulk density (4.5 g cm^**−**3^). For comparison, control cells were also assembled using a graphite anode and a NCM622 cathode. Figure 6e shows the cycling performance of the NCM622//Sn/DGT cell, displaying a gravimetric energy density of 590 W h Kg^**−**1^ with a capacity retention of 93% over 200 cycles (based on the total mass of electrode materials), which is significantly higher than that of the NCM622//graphite cell. In this cell configuration, the areal capacity of the electrode is 3.2 mA h cm^**−**2^ and the thickness of the cell (including the anode, cathode, current collector, and separator) is ~92 µm (Supplementary Fig. 23a). The volumetric energy density of the cell is estimated to be 1252 W h L^**−**1^ (Supplementary Fig. 23b). This value represents a near two-fold increase from that of the commercial NCM622//graphite cell calculated based on the same metric (647 W h L^**−**1^). Note that the current-state-of-art commercial LIBs (e.g., LIBs for microelectronics and electric vehicles) generally possess a volumetric energy density from 600 W h L^**−**1^ to 700 W h L^**−**1^, which is consistent with that of the control cell. This comparison further confirms the feasibility of fabricating LIBs with significantly improved volumetric energy density using Sn/DGT as the anodes. It is also worth noting that the volumetric energy density of the NCM622//Sn/DGT cell is still around 1.8 times higher than that of the NCM622//graphite cell (602 W h L^**−**1^) after 200 cycles (Fig. 6f). The ability to fabricate full cells with such high gravimetric and volumetric energy density, as well as long cycling life, indeed opens a new avenue towards high-performance LIBs for microelectronic and electrical vehicle application.

## Discussion

In summary, we have developed Sn anodes with both high gravimetric and volumetric capacities. This is achieved simply by encapsulating Sn nanoparticles, a metal with high gravimetric and volumetric capacity, within highly conductive and robust graphene tubes. Despite their large-volume change during cycling, the Sn nanoparticles are confined with the graphene tubes, ensuring an outstanding rate performance and long cycling life. Meanwhile, through creating the double-graphene-tubes with biphilic nature, we minimized the amount of free Sn nanoparticles in the electrodes, which could have caused rapid capacity decay upon detaching from the graphene tubes. This strategy significantly improves the gravimetric energy density and volumetric energy density of LIBs. We expect that adopting such Sn anodes could potentially double the volumetric energy density of the LIBs in the current market. Meanwhile, the selective-growth strategy can be extended to synthesize a variety of functional materials for a broad range of applications.

## Methods

### Preparation of Sn/DGT

A solution of magnesium acetate was made by adding 12 g magnesium acetate to 80 mL distilled water. A solution of urea made by adding 1.2 g urea to 20 mL water was then dropped into the magnesium acetate solution and stirred for 1 h. Subsequently, the above solution was sealed in a 200 mL Teflon-lined autoclave and maintained at 180 °C for 2 h. As-formend Mg(OH)_2_ was filtrated, washed with ethanol, dried in vacuum at 100 °C, and calcined at 600 °C for 6 h in air to obtain the MgO template.

The chemical vapor deposition (CVD) was conducted by placing the template in a quartz boat in a horizontal quartz tube. Under a flow of Ar (1000.0 mL min^**−**1^) and H_2_ (300.0 mL min^**−**1^), the reactor was heated to 900 °C, and another Ar stream (150.0 mL min^**−**1^) flowing through a flask of acetonitrile was flowed into the system for 10 min to obtain nitrogen-doped graphene coating on the template. As-formed N-doped graphene was then dispersed in 100 mL deionized water, which contain 0.5 g of Mg(NO_3_)_2_‧2H_2_O and 0.2 g of urea, and sonicated for 30 min. Then, the mixture was refluxed at 90 °C for 24 h, washed and dried at 80 °C overnight. As-obtained product was coated with graphene using CH_4_ (400.0 mL min^**−**1^) as the precursor at 1000 °C for 10 min. Finally, the obtained samples were washed using hydrochloric acid solution (1 mol L^**−**1^) to obtain the double-graphene tubes (DGT).

The encapsulation of the Sn nanoparticles within the graphene tubes was achieved following the following steps. 60 mg DGT was added in 60 mL of distilled water and sonicated for 30 min, in which 0.5 g K_2_SnO_3_·3H_2_O was dissolved and stirred for 1 h. The solution was then transferred to a 100 mL Teflon-lined autoclave, which was maintained at 180 °C for 12 h. As-formed samples were obtained by centrifugation and filtration, dried under 80 °C overnight, and annealed at 650 °C in a gas mixture of Ar (95%)/H_2_ (5%) for 10 h.

### Preparation of Sn/C

The carbon-coated Sn nanoparticles were prepared by the polymerization of dopamine-hydrochloride on commercial Sn particles with a weight ratio of 2:1 (Sn:C), and followed by the carbonization at 750 °C for 6 h^17^.

### Material characterization

The morphology and structure of the as-prepared products were conducted by field-emission scanning electron microscopy (FESEM, FEI Nova 430), transmission electron microscopy and high-resolution transmission electron microscopy (HRTEM, FEI Titan STEM). Powder X-ray diffraction was measured on Rigaku Miniflex II diffractometer with Cu Kα radiation operated at 30 kV and 15 mA. Raman spectroscopy was performed with Renishaw 2000 System. The TGA was determined on an SDT Q600 thermoanalyzer under air. In situ TEM was carried out using a FEI Titan microscope operated at 300 kV.

### Electrochemical measurement

The samples were mixed with carboxymethyl cellulose (CMC) binder and Super P carbon black to obtain uniform slurry at the weight ratio of 8:1:1. Then the slurries were coated onto copper foil and dried in a vacuum oven under 120 °C for 24 h. The electrodes were cut into a round shape with a diameter of 1.2 cm circular pieces. To measure the performance, CR2025 type coin cells were assembled using Li metal foil as the counter electrode and 1 M LiPF_6_ in ethyl carbonate/dimethyl carbonate (1:1 v/v) as electrolyte.

The charge-discharge properties were measured using Land Battery Test System (LAND CT2001A) within a voltage window from 0.01 to 2.5 V (versus Li^+^/Li) at room temperature. Electrochemical impedance measurement was evaluated in a range of 0.01–10 MHz at 10 mV with perturbation amplitude of 10 mV on the cells at open circuit potential. The capacities were calculated according to the total weight of the composites (Sn and DGT). For the full-cell performance, a commercial lithium-nickel cobalt manganese oxide (NCM622, Tianjin B&M Science and Technology Co., Ltd) was used as the cathode with a fixed areal capacity of 3.2 mA h cm^**−**2^. The N/P ratio, defined by total capacity ratio between anode and cathode, was chosen to be 1.0–1.1. The capacities were calculated according to the electrode mass loading, including the binder and carbon black.

## Supplementary information

Supplementary Information

Description of Additional Supplementary Files

Supplementary Movie 1

Supplementary Movie 2

## Data Availability

The data supporting the findings of this work are available within the article and its Supplementary Information files. All other relevant data supporting the findings of this study are available from the corresponding author on request.
