# Supplementary material for: Tin-graphene tubes as anodes for lithium-ion batteries with high volumetric and gravimetric energy densities
Source: Nat Commun. 2020 Mar 13;11:1374. doi: 10.1038/s41467-020-14859-z (PMC7069972; doi:10.1038/s41467-020-14859-z)
Supplement: Supplementary file 1 — Supplementary Information [file 41467_2020_14859_MOESM1_ESM.pdf]

Tin-graphene tubes as anodes for lithium-ion batteries with high volumetric and gravimetric energy densities

Mo et al.

Supplementary Figures

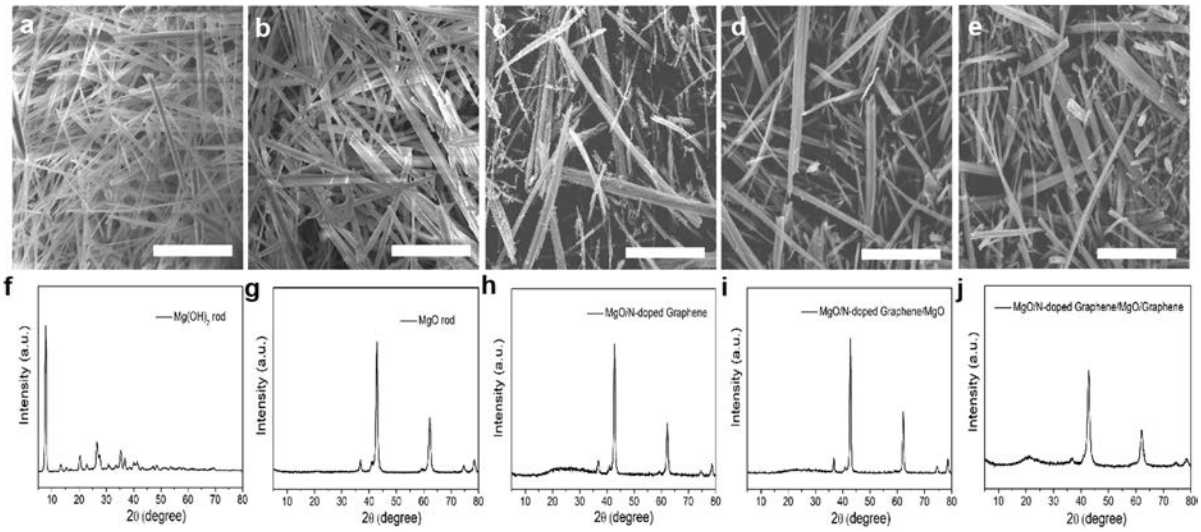

Supplementary Figure 1 **a** SEM image and **f** XRD pattern of $Mg(OH)_2$ rods. Scale bars: **a** 20 μm. **b** SEM image and **g** XRD pattern of MgO rods. Scale bars: **b** 20 μm. **c** SEM image and **h** XRD pattern of MgO/N-doped graphene. Scale bars: **c** 20 μm. **d** SEM image and **i** XRD pattern of MgO/N-doped graphene/MgO. Scale bars: **d** 20 μm. **e** SEM image and **j** XRD pattern of MgO/N-doped graphene/MgO/graphene. Scale bars: **e** 20 μm.

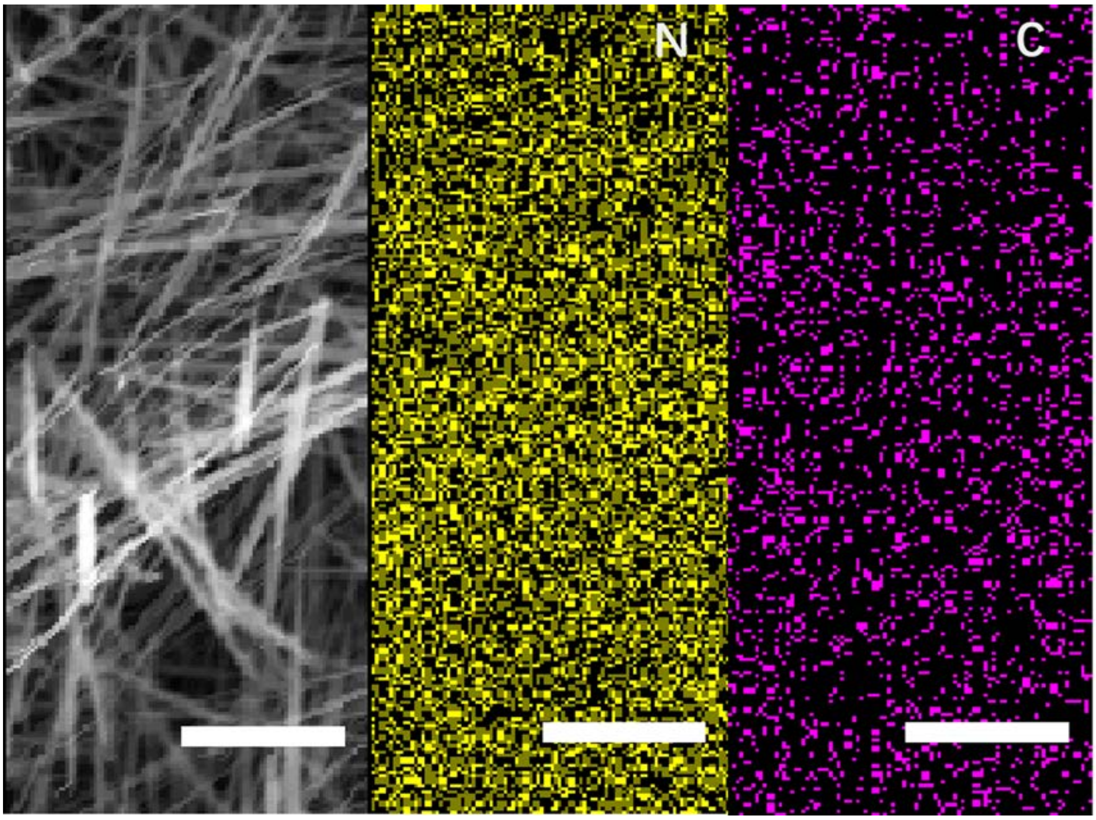

Supplementary Figure 2 EDS elemental maps of C and N. Scale bars: 5 μm.

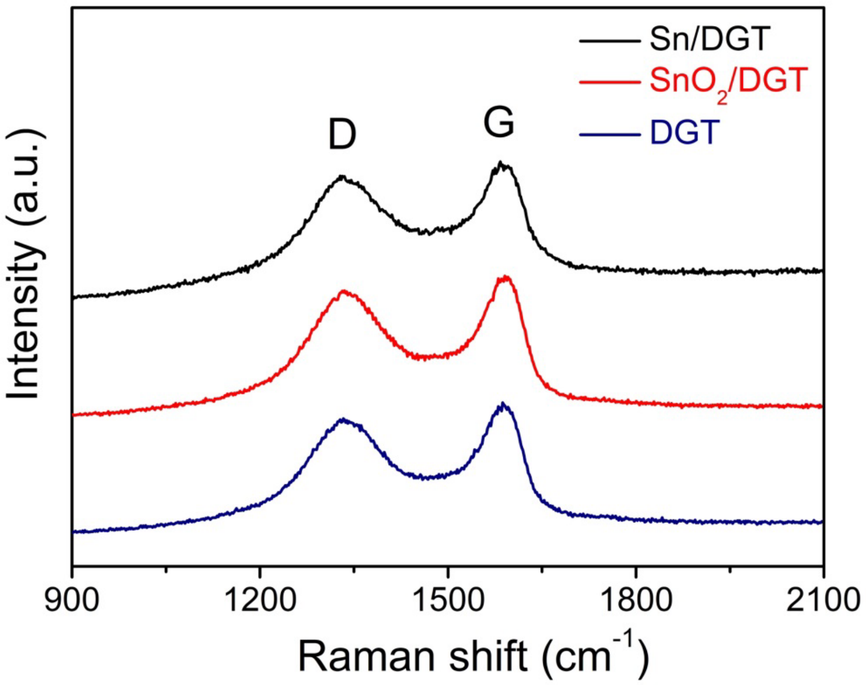

Supplementary Figure 3 Raman spectrum of DGT, SnO_2/DGT, and Sn/DGT.

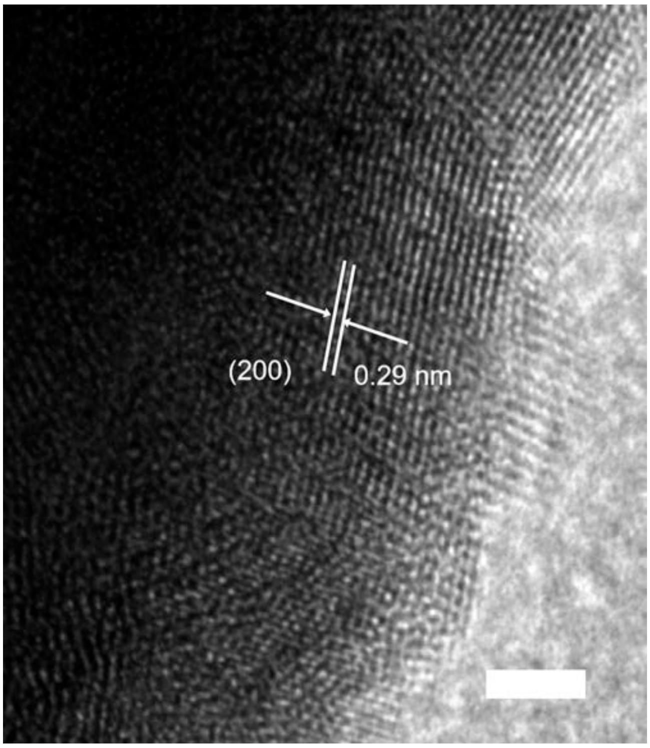

Supplementary Figure 4 High-resolution TEM image of a Sn nanoparticle in Sn/DGT. Scale bars: 2 nm.

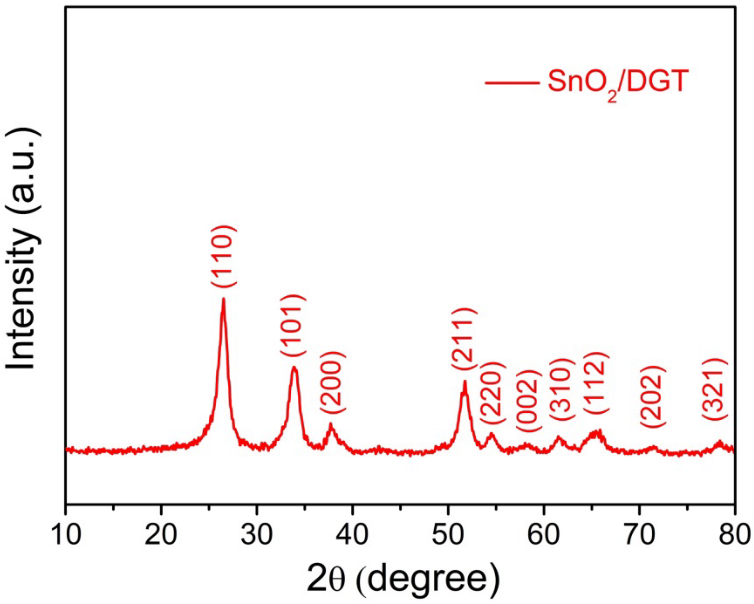

Supplementary Figure 5 XRD pattern of SnO_2/DGT

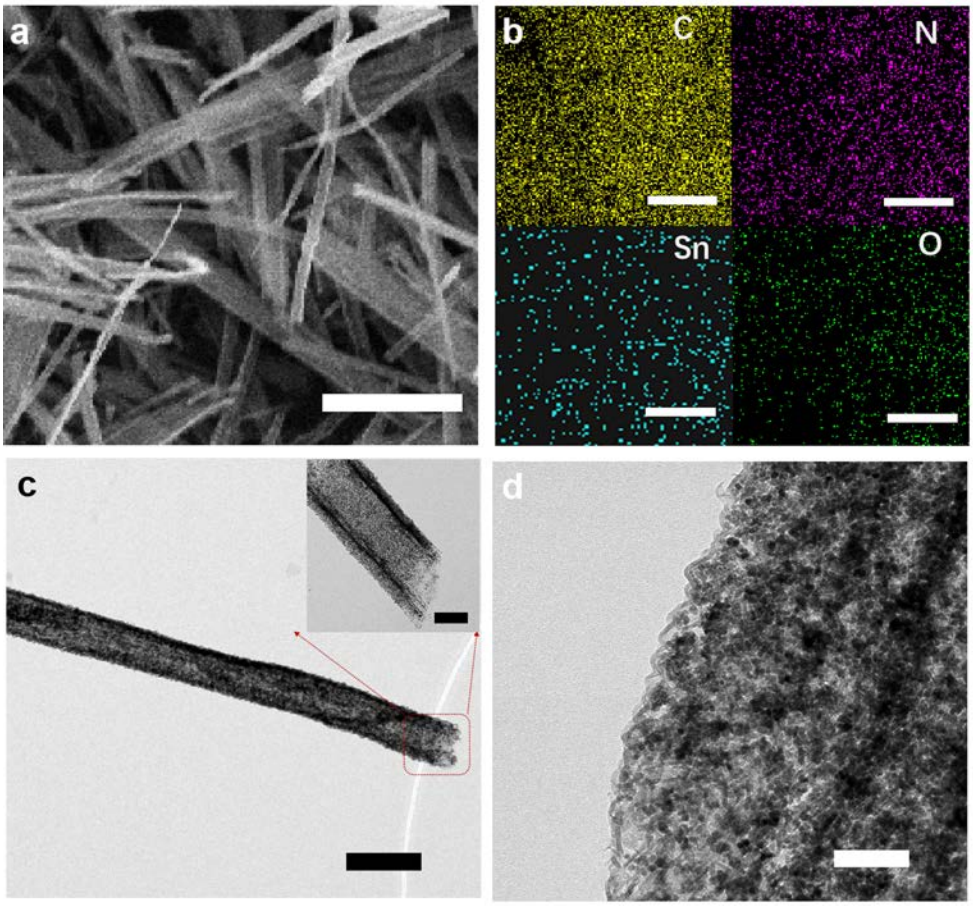

Supplementary Figure 6 a SEM image of SnO_2/DGT. Scale bars: **a** 5 μm. **b** EDS elemental maps of Sn, C, O and N. Scale bars: **b** 5 μm. **c,d** TEM images of SnO_2/DGT. Scale bars: **c** 500 nm, inset of c, 200 nm; **d** 50 nm.

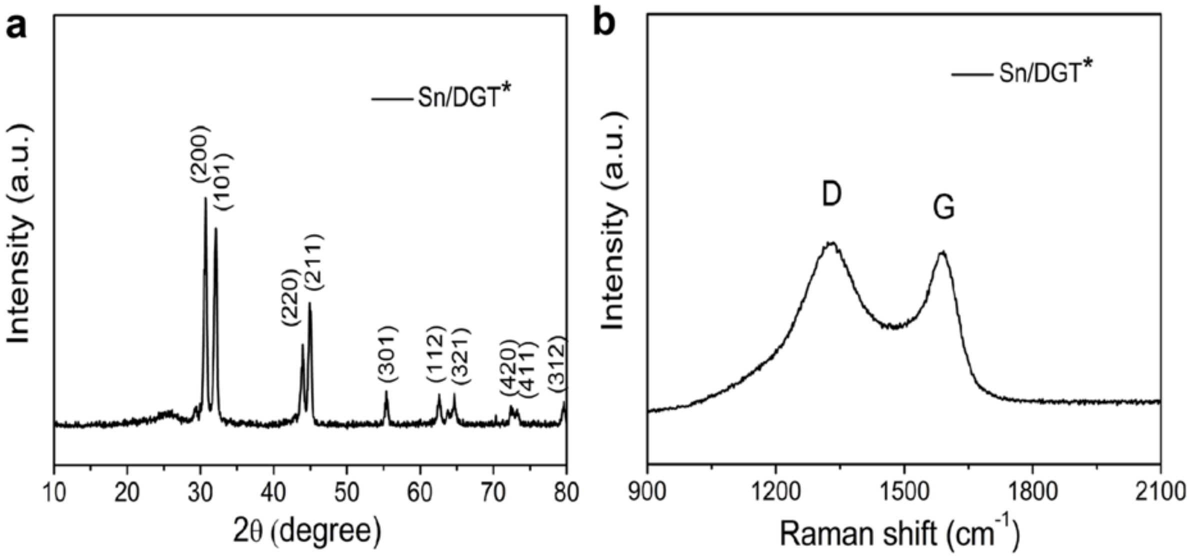

Supplementary Figure 7 a XRD pattern of Sn/DGT*. **b** Raman spectrum of Sn/DGT*.

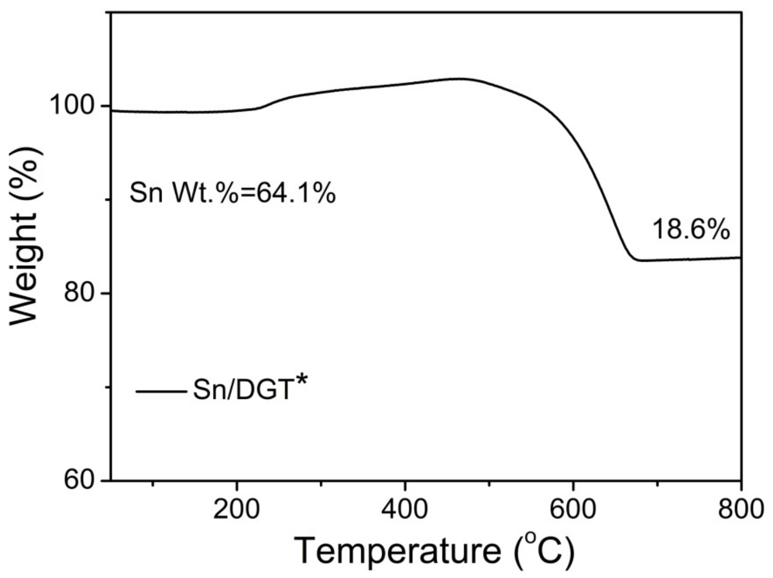

Supplementary Figure 8 TGA of Sn/DGT*.

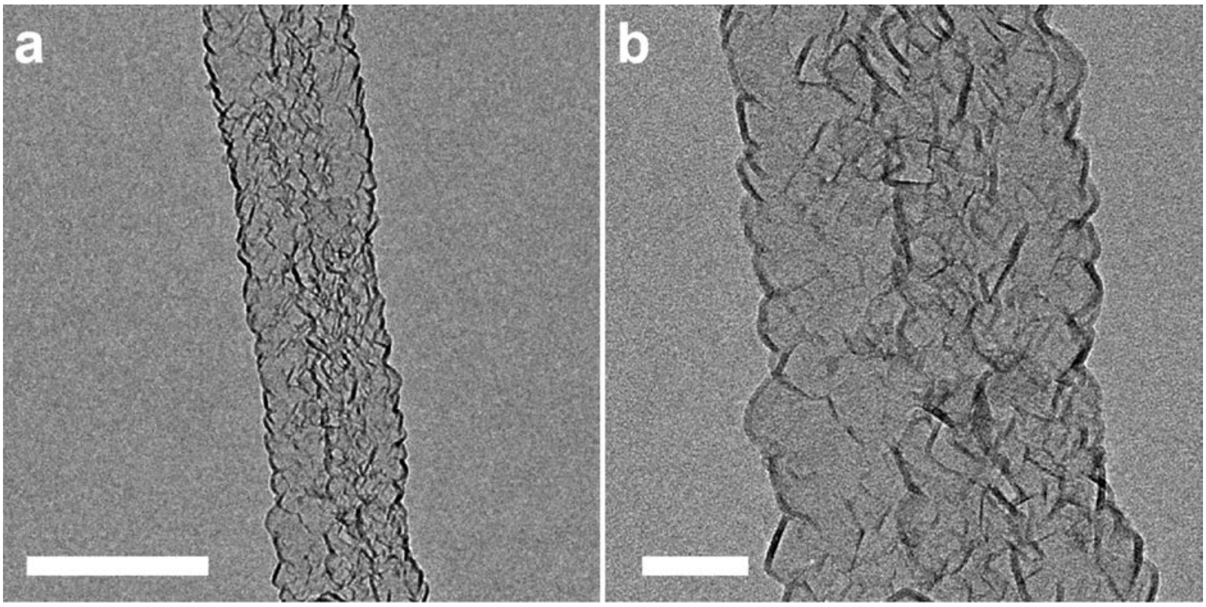

Supplementary Figure 9 TEM images of Sn/hydrophobic graphene tubes. Scale bars: **a** 500 nm; **b** 100 nm.

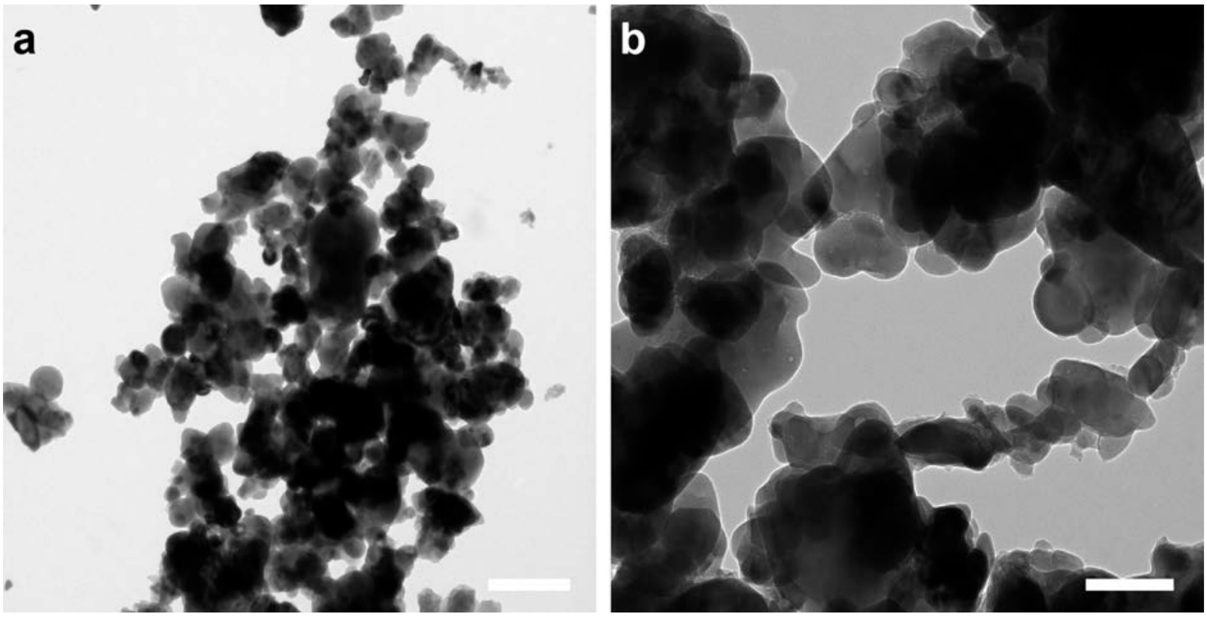

Supplementary Figure 10 a,b TEM images of the commercial Sn particles used. Scale bars: **a** 0.5 μm; **b** 200 nm.

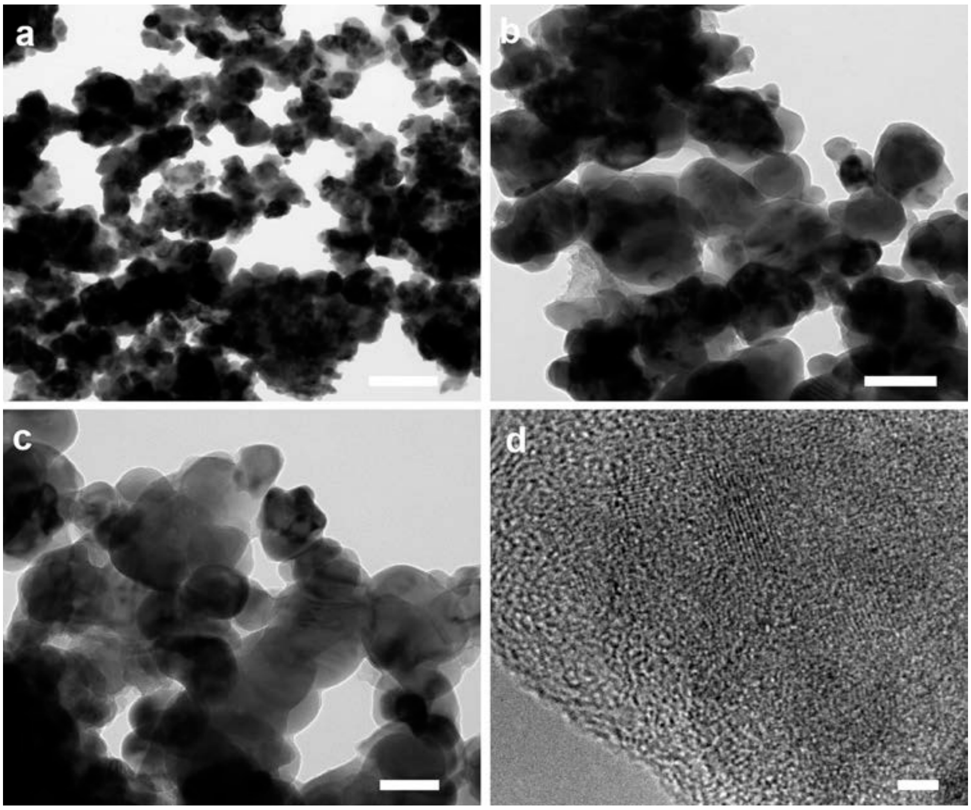

Supplementary Figure 11 TEM images of Sn/C composite. Scale bars: **a** 0.5 μm; **b** 200 nm; **c** 100 nm; **d** 2 nm.

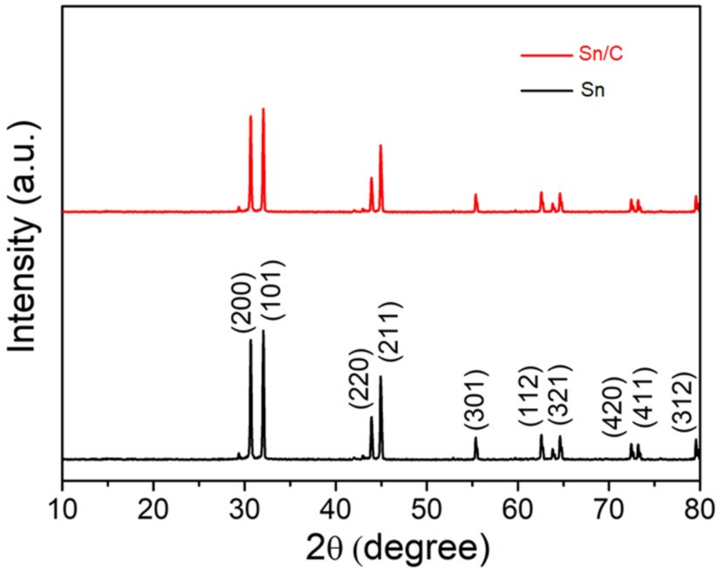

Supplementary Figure 12 XRD patterns of the commercial Sn particles and Sn/C composite.

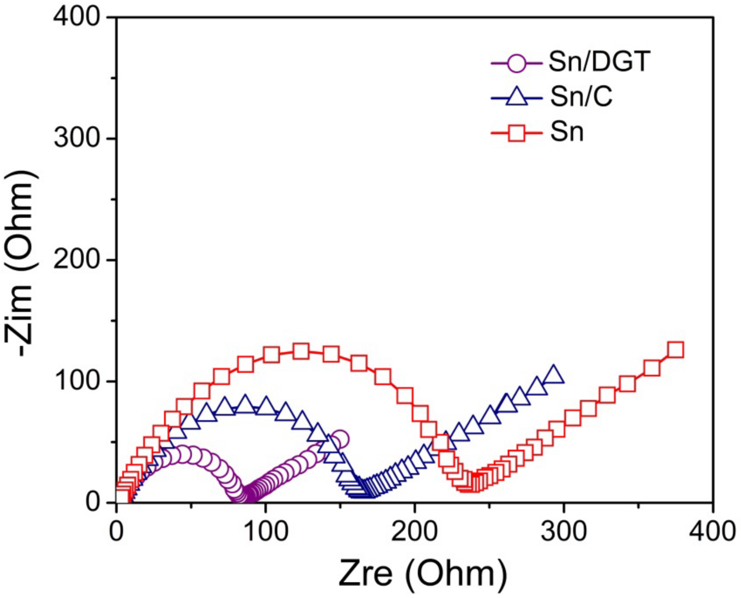

Supplementary Figure 13 Impedance spectra of Sn/DGT, Sn/C and Sn electrodes.

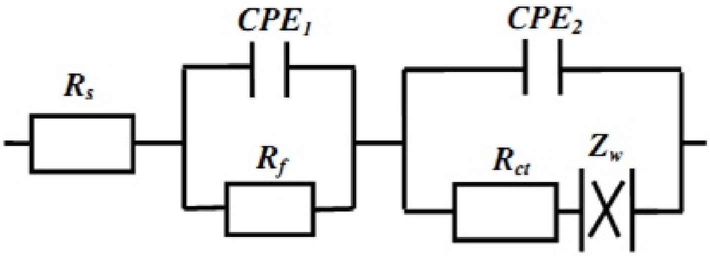

Supplementary Figure 14 An equivalent circuit proposed for the impedance behavior of the electrode.

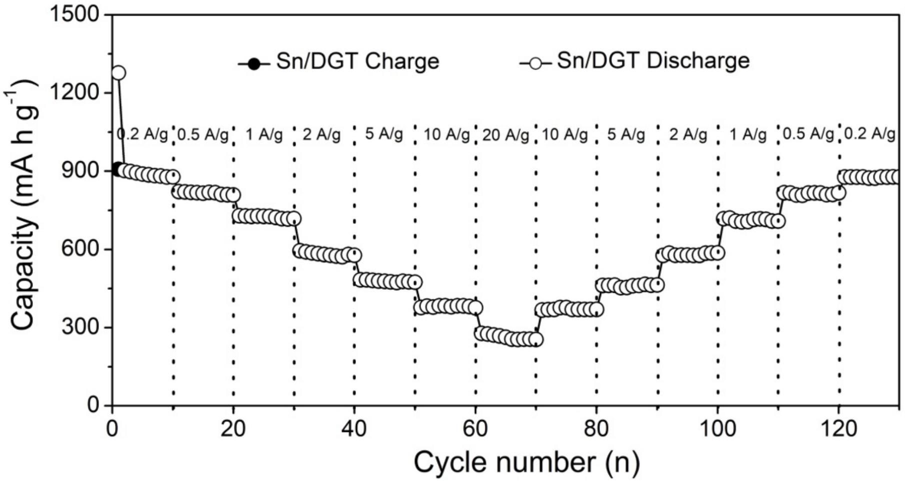

Supplementary Figure 15 The capacity of the Sn/DGT electrode fabricated without carbon black at different current densities.

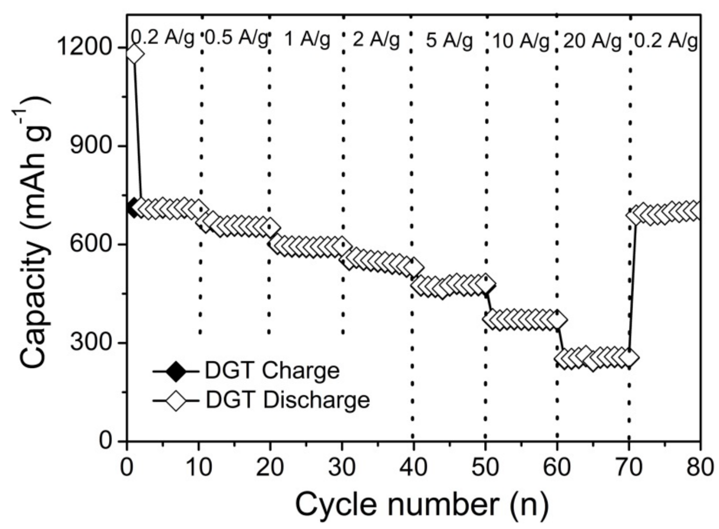

Supplementary Figure 16 The capacity of the DGT electrode at different current densities.

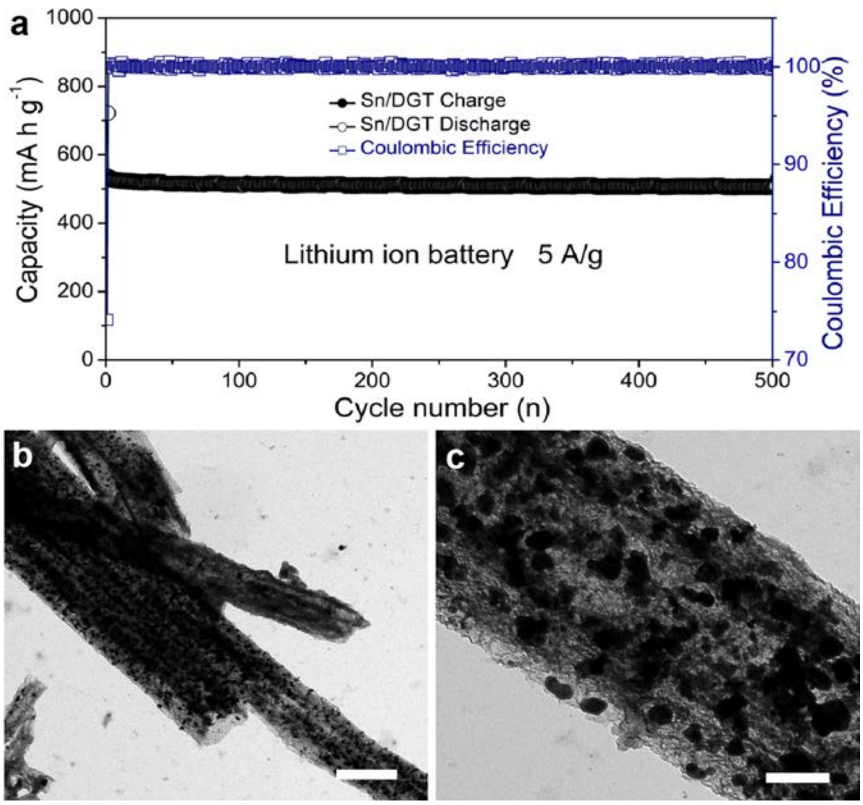

Supplementary Figure 17 The cycling stability and morphological change of Sn/DGT after 500 cycles. **a** Cycling stability of the Sn/DGT electrode at current density of 5 A g^{-1} for 500 cycles. **b,c** TEM images of the Sn/DGT electrode after 500 cycles at 5 A g^{-1}. Scale bars: **b** 1 μm; **c** 200 nm.

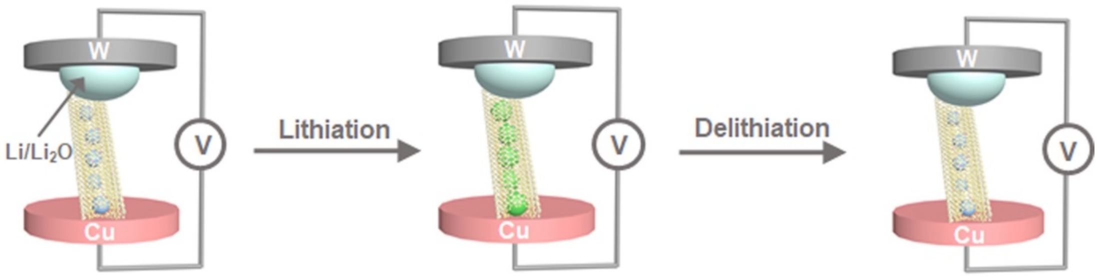

Supplementary Figure 18 A schematic of the *in situ* TEM device for observing the lithiation-delithiation process of Sn/DGT.

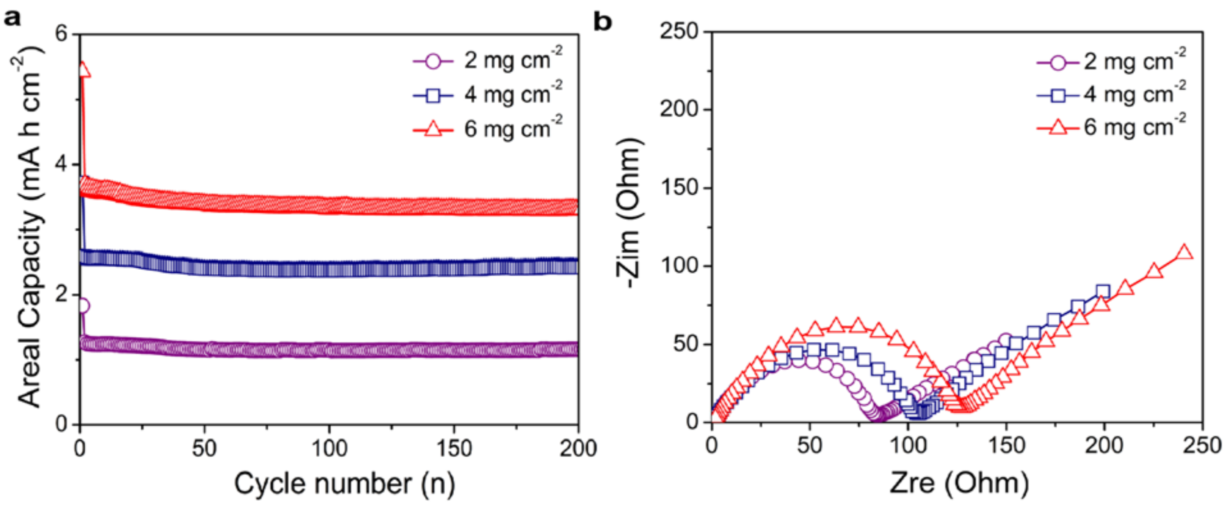

Supplementary Figure 19 a Areal capacity and cycling performance of the Sn/DGT electrode at a current density of 2 A g^{-1} for 200 cycles under different mass loadings (2, 4, and 6 mg cm^{-2}). **b** Nyquist plots of the Sn/DGT electrodes under different mass loadings (2, 4, and 6 mg cm^{-2}).

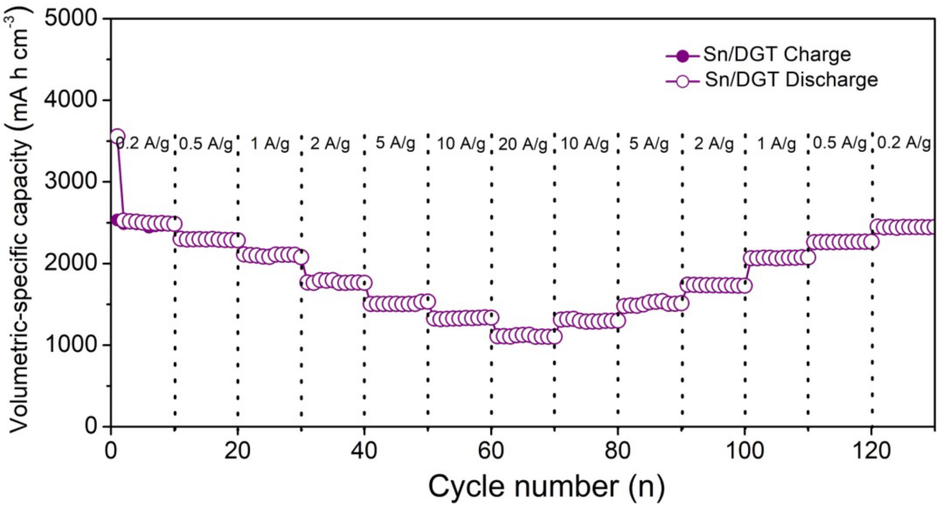

Supplementary Figure 20 The volumetric capacity of Sn/DGT electrode (active material only) at different current densities.

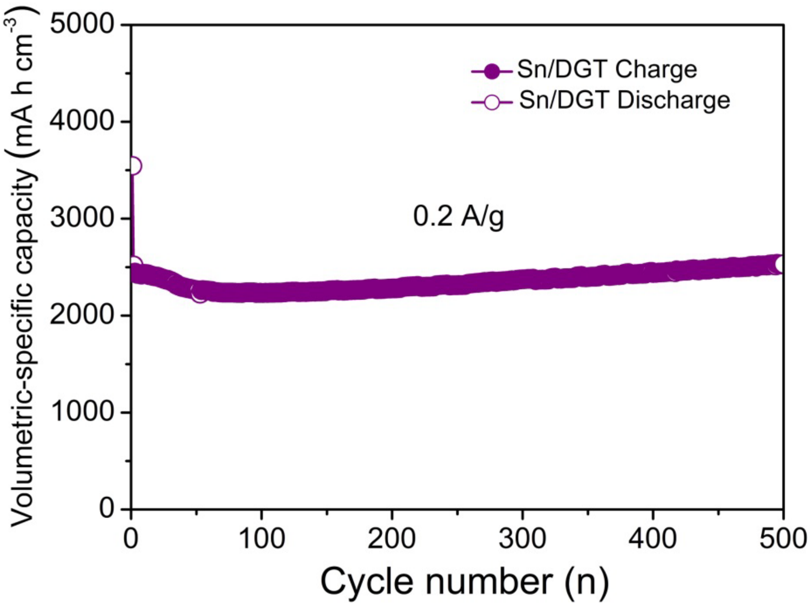

Supplementary Figure 21 Cycling performance of the Sn/DGT electrode.

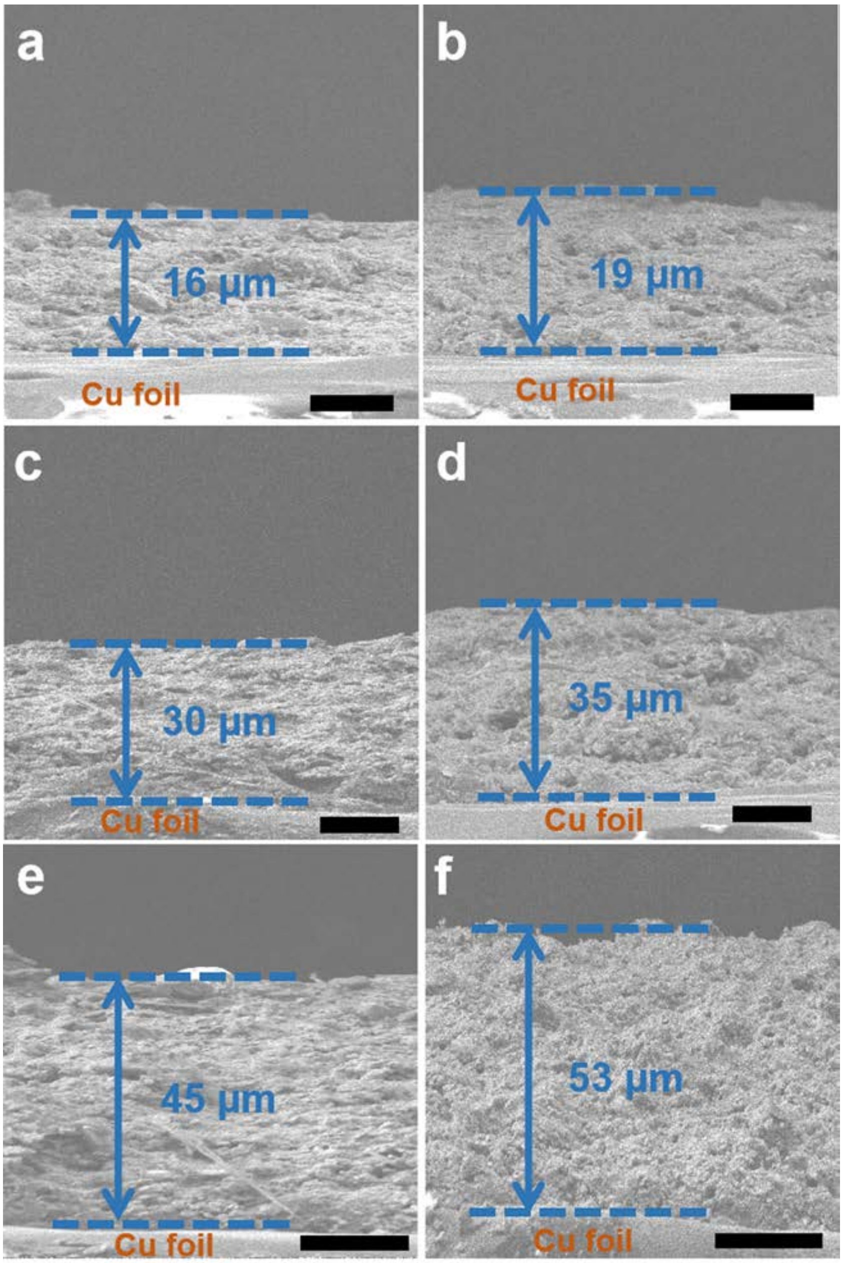

Supplementary Figure 22 Electrode thickness change before and after lithiation. **a, c, e** Cross-sectional view of the Sn/DGT electrodes with mass loading of 2, 4 and 6 mg cm^{-2} before and **b, d, f** after lithiation, respectively. Scale bars: **a** 10 μm; **b** 10 μm; **c** 15 μm; **d** 15 μm; **e** 20 μm; **f** 20 μm.

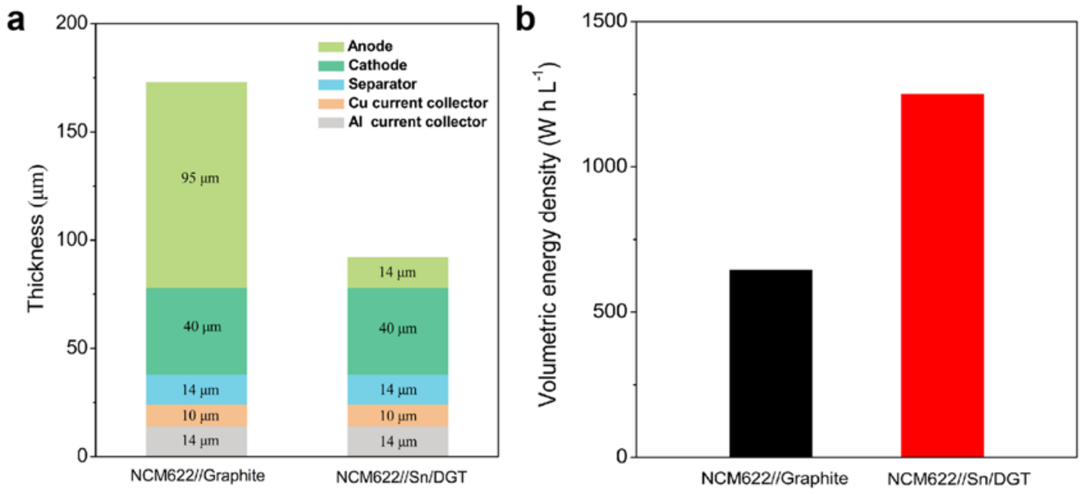

Supplementary Figure 23 **a** The electrode thickness of a NCM622//Sn/DGT and NCM622//Graphite full cell. **b** The volumetric energy density of a NCM622//Sn/DGT and a NCM622//Graphite full cell. The thickness of one-side coating on the current collector is 20 μm and 47.5 μm for the cathodes and anodes, respectively. We also assume that the electrolytes were absorbed within the electrodes and the separator without occupying an extra space. The volumetric energy density is estimated using a 3.7 V and 3.6 V for the NCM622//Graphite and NCM622//Sn/DGT cell, respectively.

Supplementary Table 1 Parameters used for the calculation based on the equivalent circuit.

	R_s (Ω)	R_f (Ω)	R_{ct}(Ω)
Sn	9	171	72
Sn/C	6	112	46
Sn/DGT	2	56	22

Supplementary Table 2 A comparison of the electrochemical performance representative Sn/carbon composites.

Materials	Sn content (wt. %)	Capacity (mA h g^{-1})	Current density (A g^{-1})	Cycles	Voltage range (V)	Ref
Graphene/Sn-nanopillar	70	608<br>408	0.1<br>5.0	30	0.002-3.0	[1]
GNS-Sn@CNT composite	34	982<br>594	0.1<br>5.0	100<br>100	0.005-3.0	[2]
Sn/C composite	58	722<br>480	0.2<br>5.0	200	0.001-2.0	[3]
Sn@G-PGNWs composite	47	1089<br>270	0.2<br>10	100	0.005-3.0	[4]
TiO_2-Sn/C core-shell nanowires	25	450<br>150	0.335<br>3.35	160<br>100	0.01-3.0	[5]
Hierarchical Sn/C composite	53	626<br>342	0.6<br>6.0	200	0.05-3.0	[6]
Sn/NC composite	42	630<br>241	0.2<br>5.0	400	0.01-3.0	[7]
F-G/Sn@C composite	57	645<br>506	0.1<br>0.4	100<br>500	0.01-2.0	[8]
Sn@ aCNT composite	67	749<br>377	0.2<br>5.0	100	0.01-2.0	[9]
Yolk-Shell Sn@C nanobox	70	810<br>350	0.2<br>4.0	500	0.005-3.0	[10]
Pipe-Wire TiO_2-Sn@CNFs	41	882<br>280	0.1<br>1.0	200	0.01-3.0	[11]
**Sn/DGT**	**71.1**	**918**	**0.2**	**500**	**0.01-2.5**	**Our work**
		**546**	**5.0**	**500**	**0.01-2.5**	
		**402**	**20.0**		**0.01-2.5**	

Supplementary Table 3 Comparison of volumetric capacity of Sn/DGT with the commercial graphite, reported representative $Li_4Ti_5O_{12}$, Sn-based, Si-based anodes in LIBs.

Materials	Density of active materials (g cm^{-3})	Volumetric capacity based on active material (mA h g^{-3})	Density of electrode (g cm^{-3})	Volumetric capacity based on the electrode (mA h g^{-3})	Density of electrode (lithiated)(g cm^{-3})	Volumetric capacity based on the electrode (lithiated) (mA h g^{-3})	Current density (A g^{-1})	Mass loading (mg cm^{-2})	Voltage range (V)	Ref
Commercial graphite	1.3	526	N/A	N/A	N/A	N/A	0.186	NA	0.01-1.5	[12]
C-$Li_4Ti_5O_{12}$ composite	1.1	195	N/A	N/A	N/A	N/A	0.175	NA	1.0-3.0	[13]
N-doped graphene	1.1	1052	N/A	N/A	N/A	N/A	0.036	2.75	0.01-3.0	[14]
Sn/C	1.92	1700	N/A	N/A	N/A	N/A	0.45	NA	0.01-3.0	[15]
PVP-Sn@ Ti_3C_2	2.16	1375	N/A	N/A	N/A	N/A	0.10	NA	0.01-3.0	[16]
SnO_2@C composite	0.24	231	N/A	N/A	N/A	N/A	0.84	NA	0.005-3.0	[17]
SnS_2@reduced graphene	1.926	1087	N/A	N/A	N/A	N/A	0.239	2.29	0.01-3.0	[18]
Si-C granule	0.49	779	N/A	N/A	N/A	N/A	2.0	NA	0.05-3.0	[19]
Si NP-PANi	0.899	1078	0.899	1078	N/A	N/A	1.0	0.3	0.01-1	[20]
3D Si membrane	0.167	429	0.167	429	N/A	N/A	0.30	NA	0.01-1.5	[21]
nC-SiMP	N/A	N/A	0.55	665	N/A	N/A	0.5	0.5	0.01-1.0	[22]
Si NW	0.233	116.5	0.233	116.5	N/A	N/A	0.179	NA	0.01-1.0	[23]
**Sn/DGT**	**2.76**	**2532**	**1.25**	**1146**	**1.05 (lithiated)**	**963 (lithiated)**	**0.20**	**2**	**0.01-2.5**	**This work**
		**2512**	**1.33**	**1210**	**1.14**	**1037**		**4**		

					(lithiated)	(lithiated)				
		2484	1.33	1197	1.13<br>(lithiated)	1017<br>(lithiated)		6		

Supplementary References

1. Ji, L. W. *et al*. Multilayer nanoassembly of Sn-nanopillar arrays sandwiched between graphene layers for high-capacity lithium storage. *Energy Environ. Sci.* **4,** 3611–3616 (2011).

2. Zou, Y. Q. & Wang, Y. Sn@CNT nanostructures rooted in graphene with high and fast Li-storage capacities. *ACS Nano*. **5**, 8108–8114 (2011).

3. Zhu, Z. Q. *et al*. Ultrasmall Sn nanoparticles embedded in nitrogen-doped porous carbon as high-performance anode for lithium-ion batteries. *Nano Lett.* **14,** 153–157 (2014).

4. Qin, J. *et al*. Graphene networks anchored with Sn@graphene as lithium ion battery anode. *ACS Nano*. **8**, 1728–1738 (2014).

5. Liao, J. Y. & Manthiram, A. A hierarchical tin/carbon composite as an anode for lithium-ion batteries with a long cycle life. *Adv. Energy Mater.* **4,** 1400403 (2014).

6. Huang, X. K. *et al*. A hierarchical tin/carbon composite as an anode for lithium-ion batteries with a long cycle life. *Angew. Chem. Int. Ed.* **54,** 1490–1493 (2015).

7. Youn, D. H., Heller, A. & Mullins, C. B. Simple synthesis of nanostructured Sn/nitrogen-doped carbon composite using nitrilotriacetic acid as lithium ion battery anode. *Chem. Mater*. **28**, 1343–1347 (2016).

8. Luo, B., Qiu, T. F., Ye, D. L., Wang, L. Z. & Zhi, L. J. Tin nanoparticles encapsulated in graphene backboned carbonaceous foams as high-performance anodes for lithium-ion and sodium-ion storage. *Nano Energy.* **22**, 232–240 (2016).

9. Zhou, X. S., Yu, L., Yu, X. Y. & Lou, X. W. Encapsulating Sn nanoparticles in amorphous

carbon nanotubes for enhanced lithium storage properties. *Adv. Energy Mater.* **6,** 1601177 (2016).

10. Zhang, H. W., Huang, X. D., Noonan, O., Zhou, L. & Yu, C. Z. Tailored yolk-shell Sn@C nanoboxes for high-performance lithium storage. *Adv. Funct. Mater.* **27,** 1606023 (2017).

11. Mao, M. L. *et al*. Pipe-wire TiO_2-Sn@carbon nanofibers paper anodes for lithium and sodium ion batteries. *Nano Lett.* **17,** 3830–3836 (2017).

12. Johnson, B. A. & White, R. E. Characterization of commercially available lithium-ion batteries. *J. Power Sources.* **70,** 48–54 (1998).

13. Jung, H. G., Kim, J. H., Scrosati, B. & Sun, Y. K. Micron-sized, carbon-coated $Li_4Ti_5O_{12}$ as high power anode material for advanced lithium batteries. *J. Power Sources.* **196,** 7763–7766 (2011).

14. Wang, X. P. *et al.* High-density monolith of N-doped holey graphene for ultrahigh volumetric capacity of Li-ion batteries. *Adv. Energy Mater*. **6**, 1502100 (2016).

15. Liu, J. Y. *et al.* High volumetric capacity three-dimensionally sphere-caged secondary battery anodes. *Nano Lett*. **16**, 4501–4507 (2016).

16. Luo, J. M. *et al*. Sn^{4+} ion decorated highly conductive Ti_3C_2 Mxene: promising lithium-ion anodes with enhanced volumetric capacity and cyclic performance. *ACS Nano*. **10**, 2491–2499 (2016).

17. Liang, J. *et al*. Bowl-like SnO_2@carbon hollow particles as an advanced anode material for lithium-ion batteries. *Angew. Chem. Int. Ed*. **53**, 12803–12807 (2014).

18. Yin, J. F., Cao, H. Q., Zhou, Z. F., Zhang, J. X. & Qu, M. Z. SnS_2@reduced graphene

oxide nanocomposites as anode materials with high capacity for rechargeable lithium ion batteries. *J. Mater. Chem*. **22**, 23963–23970 (2012).

19. Magasinski, A. *et al*. High-performance lithium-ion anodes using a hierarchical bottom-up approach. *Nat. Mater*. **9**, 353–358 (2010).

20. Wu, H. *et al*. Stable Li-ion battery anodes by in-situ polymerization of conducting hydrogel to conformally coat silicon nanoparticles. *Nat. Commun*. **4**, 1943 (2013).

21. Xia, F. *et al*. Facile synthesis of free-Standing silicon membranes with three-dimensional nanoarchitecture for anodes of lithium ion batteries. *Nano Lett*. **13**, 3340–3346 (2013).

22. Lu, Z. D. *et al*. Nonfilling carbon coating of porous silicon micrometer-sized particles for high- performance lithium battery anodes. *ACS Nano.* **9**, 2540–2547 (2015).

23. Chockla, A. M. *et al*. Silicon nanowire fabric as a lithium ion battery electrode material. *J. Am. Chem. Soc*. **133**, 20914–20921 (2011).
