# Supplementary material for: Tin-graphene tubes as anodes for lithium-ion batteries with high volumetric and gravimetric energy densities
Source: Nat Commun. 2020 Mar 13;11:1374. doi: 10.1038/s41467-020-14859-z (PMC7069972; doi:10.1038/s41467-020-14859-z)
Supplement: Supplementary file 2 — Description of Additional Supplementary Files [file 41467_2020_14859_MOESM2_ESM.pdf]

Description of Additional Supplementary Files

File Name: Supplementary Movie 1

Description: Lithiation of Sn/DGT to observe the structural stability and morphology evolution.

File Name: Supplementary Movie 2

Description: Delithiation of Sn/DGT to observe the structural stability and morphology evolution.
